# Probiotic *Bifidobacterium lactis* V9 Regulates the Secretion of Sex Hormones in Polycystic Ovary Syndrome Patients through the Gut-Brain Axis

**DOI:** 10.1128/mSystems.00017-19

**Published:** 2019-04-16

**Authors:** Jiachao Zhang, Zhihong Sun, Shuaiming Jiang, Xiaoye Bai, Chenchen Ma, Qiannan Peng, Kaining Chen, Haibo Chang, Tuanyu Fang, Heping Zhang

**Affiliations:** a College of Food Science and Technology, Hainan University, Haikou, People’s Republic of China; b Key Laboratory of Dairy Biotechnology and Engineering, Ministry of Education, Key Laboratory of Dairy Product Processing, Ministry of Agriculture, Inner Mongolia Agricultural University, Hohhot, People’s Republic of China; c Hainan Provincial People's Hospital, Haikou, People’s Republic of China; Pacific Northwest National Laboratory

**Keywords:** Bifidobacterium lactis V9, brain-gut axis, intestinal microbiome, polycystic ovary syndrome, probiotics, short-chain fatty acid

## Abstract

Although a few studies have investigated the intestinal microbiota of women with polycystic ovary syndrome (PCOS), the functional and metabolic mechanisms of the microbes associated with PCOS, as well as potential microbial biomarkers, have not yet been identified. To address this gap, we designed a two-phase experiment in which we performed shotgun metagenomic sequencing and monitored the metabolic parameters, gut-brain mediators, and sex hormones of PCOS patients. In the first stage, we identified an imbalance in the intestinal microbiota of the PCOS patients, observing that *Faecalibacterium*, *Bifidobacterium*, and *Blautia* were significantly more abundant in the control group, whereas *Parabacteroides* and *Clostridium* were enriched in the PCOS group. In the second stage, we monitored the impact of the probiotic Bifidobacterium lactis V9 on the intestinal microbiome, gut-brain mediators, and sex hormones of 14 PCOS patients. Notably, we observed that the levels of luteinizing hormone (LH) and LH/follicle-stimulating hormone (LH/FSH) decreased significantly in 9 volunteers, whereas the levels of sex hormones and intestinal short-chain fatty acids (SCFAs) increased markedly. In contrast, the changes in the indices mentioned above were indistinct in the remaining 5 volunteers. The results of an analysis of the number of viable Bifidobacterium lactis V9 cells in the two groups were highly consistent with the clinical and SCFA results. Therefore, effective host gut colonization of the probiotic Bifidobacterium lactis V9 was crucial for its ability to function as a probiotic. Finally, we propose a potential mechanism describing how probiotics regulate the levels of sex hormones by manipulating the intestinal microbiome in PCOS patients.

**IMPORTANCE** Polycystic ovary syndrome (PCOS) is a common metabolic disorder among women of reproductive age worldwide. Through a two-phase clinical experiment, we first revealed an imbalance in the intestinal microbiome of PCOS patients. By binning and annotating shotgun metagenomic sequences into metagenomic species (MGS), 61 MGSs were identified as potential PCOS-related microbial biomarkers. In the second stage, we monitored the impact of the probiotic Bifidobacterium lactis V9 on the intestinal microbiota, metabolic parameters, gut-brain mediators, and sex hormones of PCOS patients. Notably, we observed that the PCOS-related clinical indices and the intestinal microbiotas of the participating patients exhibited an inconsistent response to the intake of the B. lactis V9 probiotic. Therefore, effective host gut colonization of the probiotic was crucial for its ability to function as a probiotic. Finally, we propose a potential mechanism by which B. lactis V9 regulates the levels of sex hormones by manipulating the intestinal microbiome in PCOS patients.

## Abstract (precis)

Polycystic ovary syndrome (PCOS) is a common metabolic disorder among women of reproductive age worldwide. Through a two-phase clinical experiment, we first revealed an imbalance in the intestinal microbiome of PCOS patients. By binning and annotating shotgun metagenomic sequences into metagenomic species (MGS), 61 MGSs were identified as potential PCOS-related microbial biomarkers. In the second stage, we monitored the impact of the probiotic Bifidobacterium lactis V9 on the intestinal microbiota, metabolic parameters, gut-brain mediators, and sex hormones of PCOS patients. Notably, we observed that the PCOS-related clinical indices and the intestinal microbiotas of the participating patients exhibited an inconsistent response to the intake of the B. lactis V9 probiotic. Therefore, effective host gut colonization of the probiotic was crucial for its ability to function as a probiotic. Finally, we propose a potential mechanism by which B. lactis V9 regulates the levels of sex hormones by manipulating the intestinal microbiome in PCOS patients.

## INTRODUCTION

The human intestinal microbiome is essential for maintaining the immune system and human health (1). In recent decades, population-scale studies have highlighted the potential role of the gut microbiome in altering the health status of the host. Emerging evidence indicates that there is a close relationship between the intestinal microbiota and various chronic metabolic diseases, including type 2 diabetes (2), liver cirrhosis (3), gout (4), colorectal cancer (5), arthritis (6), and even brain disorders such as Alzheimer’s disease (7, 8) and Parkinson’s disease (9). Accordingly, severe alterations in the gut microbial structure and numerous microbial biomarkers specific to these chronic diseases have gradually been discovered. Furthermore, treatment strategies targeting patient intestinal microbiomes have been considered and represent a new approach in disease diagnosis and treatment.

Polycystic ovary syndrome (PCOS) is a common endocrine-metabolic disorder among women of reproductive age with a worldwide prevalence of 4% to 21%, depending on the diagnostic criteria used (10). PCOS is a typical metabolic disease correlated with multiple physical risk factors, including obesity, hypertension, dyslipidemia, and insulin resistance (11). Most studies on PCOS have focused on metabolic parameters and sex hormone levels, although a few studies have focused on the relationship between PCOS and the intestinal microbiota using 16S rRNA sequencing analyses (12–14). However, studies using multi-omics technologies, including deep shotgun metagenomic sequencing, are urgently needed to explore the microbial functional and metabolic mechanisms underlying PCOS and to identify potential strain-specific biomarkers for this disease.

The efficacy of probiotics in regulating and rebuilding the host intestinal microbiome is well recognized, and the probiotic interference strategy has been used to prevent and cure various metabolic diseases, including type 2 diabetes (15), inflammatory bowel disease (IBD)/irritable bowel syndrome (IBS) (16), and hyperglycemia (17). Recently, the beneficial effects of probiotic supplementation on markers for insulin metabolism and some lipid profiles in women with PCOS were reported (18). Consequently, we hypothesized that the use of probiotics could relieve the symptoms of PCOS in patients through the regulation of the intestinal microbiome.

To address these issues, we designed a cohort experiment that included two phases. In the first stage, the taxonomic structure of the intestinal microbiota in the participants was determined via high-throughput sequencing of the bacterial 16S rRNA genes. In addition, investigation of the functional profiles of the corresponding microbiomes and identification of the significantly different microbes were performed by deep shotgun metagenomic sequencing. Additionally, disease-related clinical indices and microbial metabolites were correlated with the specific biomarkers. In the second stage, we monitored the impact of probiotic supplementation on the intestinal microbiome of PCOS patients by the use of Bifidobacterium lactis V9, which was previously isolated from the gastrointestinal (GI) tract of a healthy Mongolian child, as this strain exhibited excellent probiotic characteristics in our previous study. The results of this study provide new insights into the pathogenesis and treatment of PCOS and offer objective and detailed data supporting the use of probiotic treatments for metabolic diseases.

## RESULTS

### Comparative analysis of clinical indices measured in the control and PCOS groups.

In the present study, clinical indices were measured in PCOS patients and healthy subjects (Table 1; see also Table S1 and Fig. S1 in the supplemental material). The average values determined for metabolic parameters, including levels of triglycerides (TGs), total cholesterol (TC), and fasting plasma glucose (FPG), in the PCOS group were 1.64 ± 0.26 mmol/liter, 4.73 ± 0.29 mmol/liter, and 5.98 ± 0.57 mmol/liter, respectively, and were significantly higher than those in the control group. Moreover, the metabolic levels of the typical gut-brain mediators peptide YY (PYY) and ghrelin reached 85.16 ± 10.11 pg/ml and 0.54 ± 0.03 ng/ml, respectively, in the control group and were significantly higher than those in the PCOS group. Additionally, we determined the levels of sex hormones, including luteinizing hormone (LH), follicle-stimulating hormone (FSH), prolactin (PRL), estradiol (E2), and testosterone (T), on day 3 of the menstrual cycle for each study subject. A much higher level of sex hormones was observed in the PCOS group than in the control group.

**TABLE 1:** The comparative analysis of clinical indices representing the control and PCOS groups^*a*^

Parameter	Values		Adjusted *P* value
	Control (*n* = 26)	PCOS (*n* = 38)	
Anthropometric			
Age (yrs ± SD)	26.69 ± 1.95	27.61 ± 3.82	0.329
HAIR-AN (no. of patients/total no. of patients)	0/26	9/38	NA
Acne (no. of patients/total no. of patients)	4/26	12/38	NA

Metabolic (mmol/liter ± SD)			
TG	1.2 ± 0.21	1.65 ± 0.26	0.017
TC	4.07 ± 0.26	4.73 ± 0.3	0.021
FPG	5.53 ± 0.31	5.98 ± 0.57	0.001

Brain-gut mediators (± SD)			
Ghrelin (ng/ml)	0.54 ± 0.03	0.25 ± 0.03	<0.001
PYY (pg/ml)	53.46 ± 16.25	85.16 ± 10.11	<0.001

Sex hormones (± SD)			
LH (IU/liter)	7.22 ± 2.45	17.96 ± 3.84	<0.001
FSH (IU/liter)	4.71 ± 0.57	6.98 ± 1.42	0.053
LH/FSH	1.57 ± 0.61	2.63 ± 0.61	<0.001
E2 (pg/ml)	52.82 ± 6.08	56.32 ± 15.02	0.166
PRL (ng/ml)	9.22 ± 1.37	11.79 ± 1.61	<0.001
T (nm/liter)	0.83 ± 0.3	6.02 ± 1.13	<0.001

SCFA (μmol/g ± SD)			
Acetic acid	57.36 ± 9.33	24.6 ± 8.94	0.008
Propionic acid	20.14 ± 5.96	13.93 ± 3.84	<0.001
Butyric acid	12.86 ± 4.2	5.05 ± 1.59	0.021
Valeric acid	1.66 ± 0.64	0.55 ± 0.3	0.003

a HAIR-AN, hyperandrogenism (HA), insulin resistance (IR), and acanthosis nigricans (AN); NA, not applicable.

10.1128/mSystems.00017-19.1 FIG S1 Comparative analysis of the clinical indices of the control and PCOS groups. Each color dot (green for the control group and pink for the PCOS group) represents the average values of the metabolic parameters, including triglycerides (TGs), total cholesterol (TC), fasting plasma glucose (FPG), sex hormone, short-chain fatty acids (SCFAs), and the typical gut-brain mediators PYY and ghrelin. Download FIG S1, TIF file, 0.2 MB. Copyright © 2019 Zhang et al. 2019 Zhang et al. This content is distributed under the terms of the Creative Commons Attribution 4.0 International license.

10.1128/mSystems.00017-19.3 TABLE S1 The clinical and body indices for all volunteers. Download Table S1, XLSX file, 0.02 MB. Copyright © 2019 Zhang et al. 2019 Zhang et al. This content is distributed under the terms of the Creative Commons Attribution 4.0 International license.

### Imbalances in the intestinal microbiotas of the PCOS patients.

**FIG 1:**
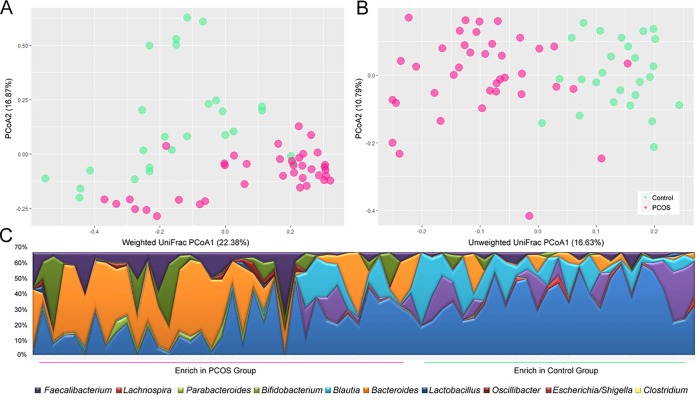
The alterations in the intestinal microbiota of the PCOS patients. (A and B) Principal-component-analysis (PCoA) score plot based on weighted (A) and unweighted (B) UniFrac metrics for all participants. Each color dot (green for the control group and pink for the PCOS group) represents the composition of the intestinal microbiota based on the results of 16S rRNA gene high-throughput sequencing performed for each subject. (C) The differences in intestinal microbes between the PCOS and control groups at the genus level. Significance was calculated by the Wilcoxon rank sum test.

For each of the 64 subjects, the organismal structure of the intestinal microbiota was analyzed by sequencing the 16S rRNA gene amplicons. A principal-coordinate analysis (PCoA) was performed based on the weighted and unweighted UniFrac distances of the 16S rRNA sequence profiles at the genus level (Fig. 1A and B). The intestinal microbiotas from the control and PCOS groups were highly distinct with respect to organismal structure, with the subjects forming two clusters that corresponded to the two experimental groups. To correct for any possible population stratification caused by non-disease-related factors that could impact the structure of intestinal microbiota, an Adonis test was performed (Table S2 and S3). After correction, the effects associated with the non-disease-related factors disappeared. For example, the *R*^2^ values which represented the correlation between the values representing certain host factors (i.e., age and HAIR-AN [hyperandrogenism {HA}, insulin resistance {IR}, and acanthosis nigricans {AN}] and acne values) and the host’s intestinal microbiota were 0.023, 0.023, and 0.011, respectively. Those values are far lower than the *R*^2^ values determined for the disease factor, further confirming that PCOS was a significant factor explaining the observed variation in the intestinal microbiota. At the genus level, we observed that the abundances of *Faecalibacterium*, *Lachnospira*, *Bifidobacterium*, and *Blautia* were significantly higher in the control group than in the PCOS group, whereas those of *Parabacteroides*, *Bacteroides*, *Lactobacillus*, *Oscillibacter*, *Escherichia/Shigella*, and *Clostridium* were enriched in the PCOS group (Fig. 1C; Wilcoxon rank sum tests).

10.1128/mSystems.00017-19.4 TABLE S2 Weighted and unweighted UniFrac distance-based variables (Adonis tests). Download Table S2, DOCX file, 0.03 MB. Copyright © 2019 Zhang et al. 2019 Zhang et al. This content is distributed under the terms of the Creative Commons Attribution 4.0 International license.

10.1128/mSystems.00017-19.5 TABLE S3 Weighted and unweighted UniFrac distance-based metagenomic species (Adonis tests). Download Table S3, DOCX file, 0.04 MB. Copyright © 2019 Zhang et al. 2019 Zhang et al. This content is distributed under the terms of the Creative Commons Attribution 4.0 International license.

**FIG 2:**
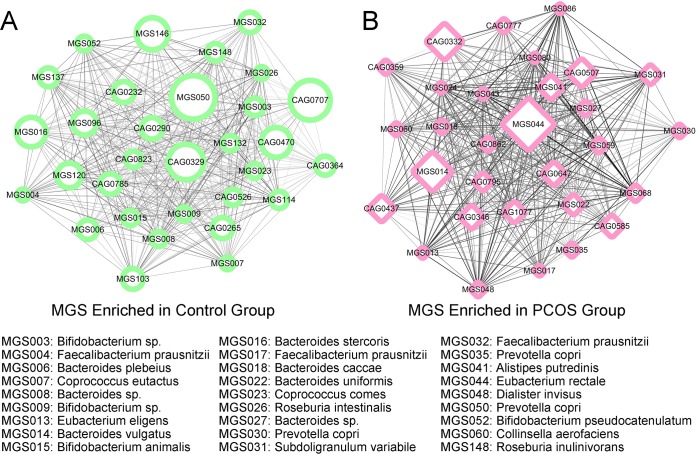
Taxonomic characterization of the intestinal microbiota (metagenomic species [MGS]) in the different groups. Differentially abundant MGS networks that were enriched in the control group (*n* = 31; panel A, green) and the PCOS individuals (*n* = 30; panel B, pink). The network was constructed based on Spearman's rank correlation coefficient values determined in comparisons of different MGS. The edge width is proportional to the correlation strength. The node size is proportional to the mean abundance in the respective population. Each node represents an MGS, and the identified MGS is annotated with its species as indicated in the footnote.

To further explore the differential intestinal microbes at the strain level, we binned the shotgun metagenomic sequencing data into 1,080 coabundance gene groups (CAGs) (Table S4), and the 151 CAGs containing more than 50 contigs were reassembled into MGS. Finally, 45 MGS were assigned specific taxonomic levels. Through a comparative analysis, we observed that 30 CAGs, including Subdoligranulum variabile MGS031, Prevotella copri MGS035, Eubacterium rectale MGS044, Eubacterium eligens MGS013, Dialister invisus MGS048, Collinsella aerofaciens MGS060, Bacteroides vulgatus MGS014, Bacteroides uniformis MGS022, *Bacteroides* sp. MGS027, Bacteroides caccae MGS018, and Alistipes putredinis MGS041, were enriched in the PCOS group (Fig. 2B), while 31 CAGs, including *Bifidobacterium* sp. MGS003, Faecalibacterium prausnitzii MGS004, *Bacteroides* sp. MGS008, Bifidobacterium animalis MGS015, Roseburia inulinivorans MGS148, Coprococcus eutactus MGS007, Bifidobacterium pseudocatenulatum MGS052, Roseburia intestinalis MGS026, Bacteroides stercoris MGS016, Coprococcus comes MGS023, Bacteroides plebeius MGS006, and Faecalibacterium prausnitzii MGS032, were enriched in the control group (Fig. 2A). Interestingly, we noted a lack of the beneficial microbes Faecalibacterium prausnitzii and *Bifidobacterium* sp., as well as the presence of the disease-related microbes Prevotella copri and Collinsella aerofaciens, in the PCOS patients.

10.1128/mSystems.00017-19.6 TABLE S4 The metagenomic species (MGS) profile for all subjects. Download Table S4, XLSX file, 0.09 MB. Copyright © 2019 Zhang et al. 2019 Zhang et al. This content is distributed under the terms of the Creative Commons Attribution 4.0 International license.

### Alterations in the annotated microbial metabolic pathways and short-chain fatty acids (SCFAs).

**FIG 3:**
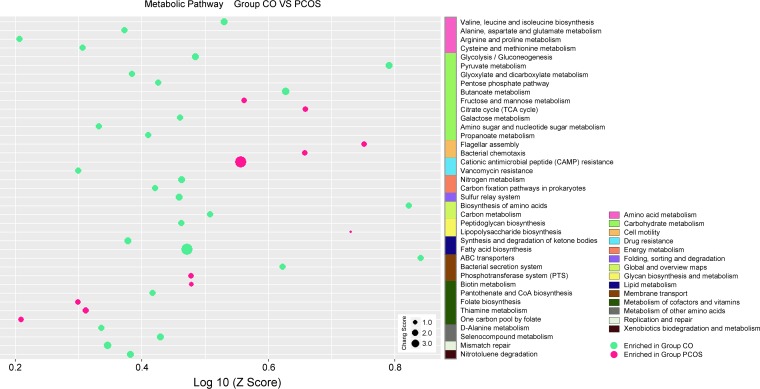
The log-transformed reporter Z scores of the metabolic pathways showing significant differences between the PCOS and control groups. The green nodes represent the metabolic pathways (in level 3) enriched in the control group (CO), and the pink nodes represent the metabolic pathways (in level 3) enriched in the PCOS group (PCOS). The node size represents the changed score. The metabolic pathways in level 2 are displayed in different colors. CoA, coenzyme A.

To investigate the observed differences in the functional profiles of the intestinal microbiota between the PCOS patients and the healthy subjects, high-quality reads from all samples were obtained and annotated for protein-coding genes. Based on the results, a collective, nonredundant intestinal microbiota gene catalogue for PCOS was created. Next, for each sample, the reads were mapped to the collective gene catalog to reconstruct sample-specific gene profiles, and profiles were also generated using the Kyoto Encyclopedia of Genes and Genomes database orthologs (KO; Table S5). Metabolic pathways associated with fructose and mannose metabolism, the citrate cycle (tricarboxylic acid [TCA] cycle), flagellar assembly, bacterial chemotaxis, cationic antimicrobial peptide (CAMP) resistance, lipopolysaccharide biosynthesis, the phosphotransferase system (PTS), biotin metabolism, folate biosynthesis, thiamine metabolism, and one carbon pool by folate were enriched in the PCOS group, whereas the metabolic pathways associated with valine, leucine and isoleucine biosynthesis, propanoate metabolism, fatty acid biosynthesis, ABC transporters, and bacterial secretion systems were enriched in the control group (Fig. 3). Accordingly, we determined the concentrations of short-chain fatty acids, including acetic, propionic, butyric, and valeric acids (Table 1; see also Fig. S1), which are considered the main intestinal microbial beneficial metabolites, in the fecal samples of the participants. Unsurprisingly, the amounts of acetic, propionic, butyric, and valeric acids in PCOS patients were 24.59 ± 8.94, 13.93 ± 3.84, 5.05 ± 1.59, and 0.55 ± 0.29 μmol/g, respectively, and were significantly lower than those in the control group.

10.1128/mSystems.00017-19.2 FIG S2 The structure of the intestinal microbiota changed markedly in the response group (group R, *n* = 9) based on the 16S rRNA gene high-throughput-sequencing weighted UniFrac distance. The dots exhibiting different shapes and different depths of green color (the green color gradually darkened during the time period of 0 to 10 weeks) represent the composition of the intestinal microbiota of each subject. Download FIG S2, TIF file, 0.4 MB. Copyright © 2019 Zhang et al. 2019 Zhang et al. This content is distributed under the terms of the Creative Commons Attribution 4.0 International license.

10.1128/mSystems.00017-19.7 TABLE S5 The KEGG profile for all subjects. Download Table S5, XLSX file, 2.3 MB. Copyright © 2019 Zhang et al. 2019 Zhang et al. This content is distributed under the terms of the Creative Commons Attribution 4.0 International license.

### Correlation analysis of the identified MGS and the metabolic parameters, SCFAs, and sex hormones.

**FIG 4:**
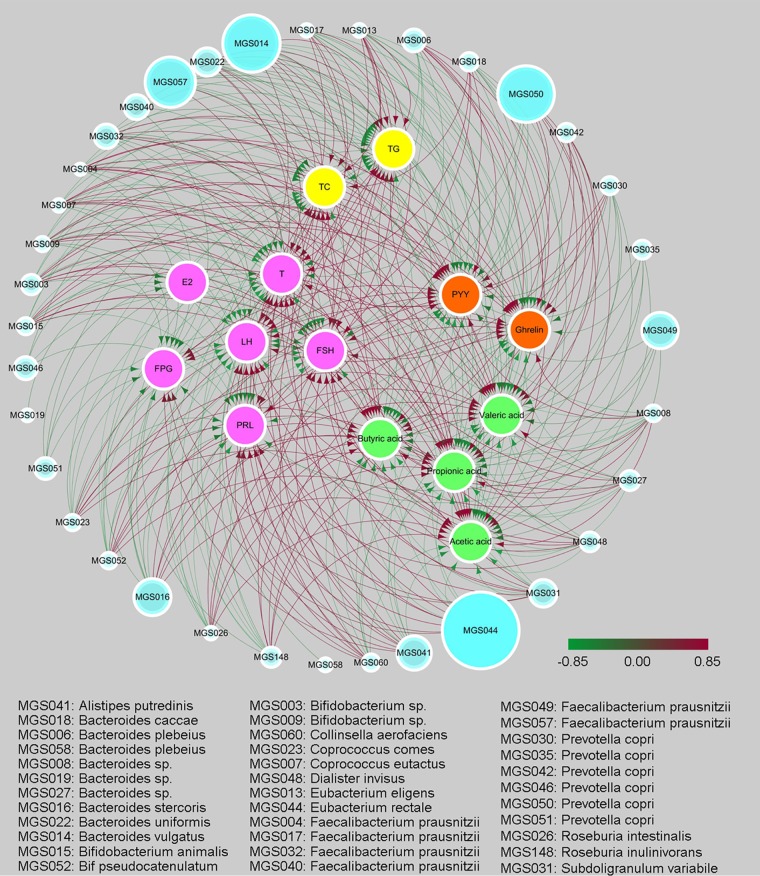
The correlation network constructed using the identified metagenomic species (MGS), metabolic parameters (including TGs, TC, and FPG), SCFAs (including acetic, propionic, butyric, and valeric acid), gut-brain mediators (PYY and ghrelin), and sex hormones (including LH, FSH, LH/FSH, E2, PRL, and T) based on Spearman's rank correlation coefficient (*R* greater than 0.4 or *R* less than −0.4). The edge width and color (red, positive; green, negative) are proportional to the strength of the correlation. The node size is proportional to the mean abundance in the respective population. Nodes with the same color are classified as the same type of indicators. Bif, *Bifidobacterium*.

We further explored the correlations among the identified MGS, metabolic parameters, SCFAs, and sex hormones by constructing a network based on the determined Spearman’s rank correlation coefficients (Fig. 4; see also Table S6). As shown in Fig. 4, a generally positive correlation was observed between Bifidobacterium animalis and Faecalibacterium prausnitzii and the SCFAs as well as the gut-brain mediators, whereas a negative correlation was observed between these two species and the sex hormone levels. In contrast, the species Coprococcus eutactus, Collinsella aerofaciens, Prevotella copri, and Bacteroides caccae were positively correlated with the levels of sex hormones but negatively correlated with the levels of SCFAs and gut-brain mediators. By performing the Adonis test on the UniFrac distance matrix data shown in Fig. 1 using the same variables listed in Fig. 4, we linked the interpretations of the 16S amplicon sequencing data and the metagenomic sequencing data (Table S2 and S3). The variables (including TGs, TC, LH, T, PYY, ghrelin, and SCFAs) and the metagenomic species (including Bifidobacterium animalis MGS015, *Bifidobacterium* sp. MGS003, and *Bifidobacterium* sp. MGS009) were closely correlated with changes in the intestinal microbiota of the PCOS patients.

10.1128/mSystems.00017-19.8 TABLE S6 The *R* values representing comparisons between the metagenomic species (MGS) and the clinical factors. Download Table S6, XLSX file, 0.03 MB. Copyright © 2019 Zhang et al. 2019 Zhang et al. This content is distributed under the terms of the Creative Commons Attribution 4.0 International license.

### Fluctuations in the levels of SCFAs, sex hormones, and signal peptides related to the colonization of Bifidobacterium lactis V9.

**FIG 5:**
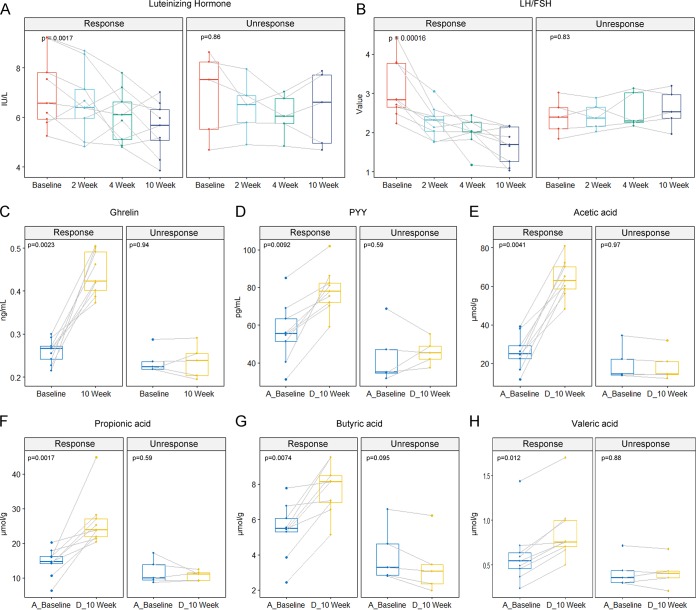
Dynamic changes in the levels of sex hormones (panel A for LH and panel B for LH/FSH), brain-gut mediators (panel C for ghrelin and panel D for PYY), and SCFAs (panels E, F, G, and H for acetic acid, propionic acid, butyric acid, and valeric acid, respectively) during the Bifidobacterium lactis V9 consumption stage. Each panel is composed of two groups: the response group (group R; the changes in the indices listed above were notable for this group) and the nonresponse group (group N; the changes in the indices listed above were limited for this group). The significance data were calculated using the Kruskal-Wallis test (for comparisons of data from multiple time points) and the Wilcoxon rank sum test (for comparisons of data from two [paired] time points). The variation trends in the indices listed above for each participant are indicated with a solid line.

After demonstrating the alterations in the intestinal microbiome in the PCOS group at the taxonomic and functional levels, we further investigated the key roles of the beneficial microbes *Bifidobacterium* and Faecalibacterium prausnitzii, which were closely correlated with PCOS. Considering its safety and universality and the results of our previous studies (19, 20), we chose the probiotic Bifidobacterium lactis V9 strain for the subsequent experiments. Fourteen PCOS patients participated in a study to investigate the impact of Bifidobacterium lactis V9 on the intestinal microbiota of individuals with PCOS. By monitoring the sex hormone, signal peptide, and intestinal SCFA levels in the 14 volunteers during the Bifidobacterium lactis V9 consumption period, we observed dramatic changes in the indices mentioned above. The levels of LH and LH/FSH decreased significantly in 9 volunteers, whereas the levels of sex hormones increased markedly, as did the intestinal SCFA levels, including the levels of acetic acid, propionic acid, butyric acid, and valeric acid (Fig. 5). In contrast, the fluctuations in these indices were not notable in the remaining 5 volunteers. Accordingly, the 14 volunteers were divided into two groups, namely, the response group (group R) and the nonresponse group (group N).

**FIG 6:**
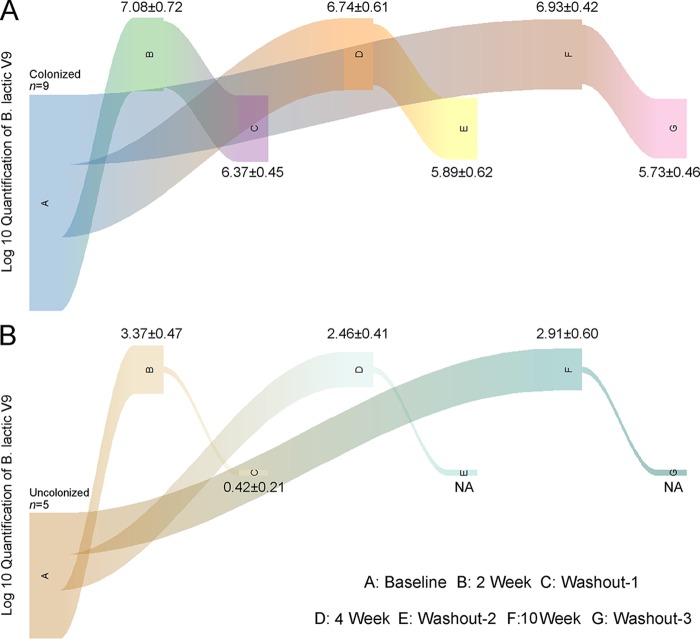
Quantification of live Bifidobacterium lactis V9 in fecal samples during the intake and washout stages for the response group (group R, *n* = 9) and the nonresponse group (group N, *n* = 5). The different experimental stages, including the baseline (point A), probiotic consumption (points B, D, and F), and washout (points C, E, and G) stages, are arranged at the bottom, the top, and the middle of the Sankey diagram. The thicknesses of the curves in different colors represent the average amounts of viable Bifidobacterium lactis V9 in fecal samples. As shown in the figure, the lines in panel A are thick, which indicates that the average amount of viable Bifidobacterium lactis V9 in group R was high, whereas the lines in panel B are thin, which indicates that the average amount of viable Bifidobacterium lactis V9 in group N was low.

Probiotics are defined as live microorganisms that confer health benefits to the gut microbiota of the host when present in adequate amounts. The host’s gastrointestinal tract is the main “battleground” where the probiotic is able to exert its health-promoting functions. Therefore, the effective colonization of a consumed probiotic in the host gut is crucial for its health-promoting characteristics. By performing quantitative PCR (q-PCR) with a strain-specific primer, we quantified the number of viable Bifidobacterium lactis V9 cells in the two groups. The results were highly consistent with the clinical and SCFA results. We observed that the average abundance of viable Bifidobacterium lactis V9 reached 6.93 ± 0.42 log CFU/g of feces in group R during the Bifidobacterium lactis V9 consumption phase (Fig. 6). More convincingly, no significant decline in the abundance of viable cells was observed during the washout phase in group R. In contrast, few live probiotic Bifidobacterium lactis V9 organisms survived in the guts of the participants in group R in both the consumption and washout phases. These results indicate a robust correlation between the abundance of the colonized probiotic Bifidobacterium lactis V9 and the PCOS-related clinical indices and intestinal SCFAs.

### Differing responses of the intestinal microbiota to the consumption of the probiotic Bifidobacterium lactis V9.

**FIG 7:**
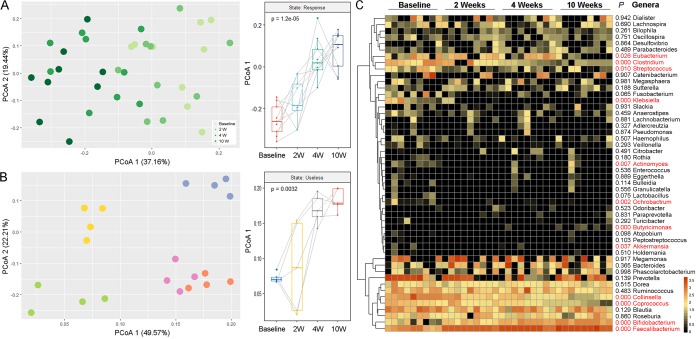
The intestinal microbiota exhibited an inconsistent response to the Bifidobacterium lactis V9 probiotic intake. (A) The structure of the intestinal microbiota changed markedly in the response group (group R, *n* = 9) based on the 16S rRNA gene high-throughput-sequencing weighted UniFrac distance. The dots exhibiting different depths of green color (the green color gradually darkened from 0 to 10 weeks) represent the composition of the intestinal microbiota of the subjects. (B) The influence of the probiotic Bifidobacterium lactis V9 on the gut microbiota in the nonresponse group (group N, *n* = 5) was limited according to the clusters based on the weighted UniFrac distance. The dots with the same color represent the composition of the intestinal microbiota of the same participant at different time points. (C) A heat map representing the intestinal microbial changes at the genus level in group R at different Bifidobacterium lactis V9 consumption stages. The degrees of depth of the color represents the relative abundances of the related genus. The *P* value was calculated for each genus by the Kruskal-Wallis test, and the data are annotated on the left side. *P* values of less than 0.05 are marked in red.

Based on the results described above, we further addressed the issue of how the intestinal microbiota responded to the intake of the probiotic Bifidobacterium lactis V9 in group R and group N. Unsurprisingly, marked changes in the structure of the intestinal microbiota were observed in group R through a principal-coordinate analysis (PCoA) based on UniFrac distance (Fig. 7A; see also Fig. S2). In this group, increased abundances of the genera *Bifidobacterium*, *Faecalibacterium*, *Butyricimonas*, and *Akkermansia* were observed in addition to decreases in the abundances of the genera *Collinsella*, *Coprococcus*, *Klebsiella*, *Clostridium*, *Actinomyces*, *Streptococcus*, *Eubacterium*, and *Ochrobactrum* (Fig. 7C; see also Table S7). In contrast, the influence of the probiotic Bifidobacterium lactis V9 on the gut microbiotas in group N was limited (Fig. 7B), and individual factors dominated the structure of the intestinal microbiotas throughout the experiment.

10.1128/mSystems.00017-19.9 TABLE S7 The genus-level abundance determined for each volunteer in stage 2. Download Table S7, XLSX file, 0.03 MB. Copyright © 2019 Zhang et al. 2019 Zhang et al. This content is distributed under the terms of the Creative Commons Attribution 4.0 International license.

## DISCUSSION

Polycystic ovary syndrome leads to several complications, including hyperandrogenism, obesity, and metabolic syndrome. In this study, in agreement with the current literature, it was shown that LH, testosterone, and PRL levels were elevated and that the levels of brain-gut mediators, including ghrelin and PYY, were lowered in PCOS patients compared to the levels in healthy controls. Ghrelin is an acylated 28-amino-acid peptide that was identified in the rat stomach, and its presence enhances appetite, reduces fat utilization, and leads to adiposity (21). PYY is colocated with GLP-1 in the L-cells of the distal gut and is involved in the so-called “ileal brake” (22). Recent observations have revealed that in addition to these known metabolic effects, ghrelin and PYY are also involved in other functions concerning the hypothalamus-pituitary gland-gonad axis (23). In line with our present results, previous researchers reported that the factors responsible for the elevated LH and testosterone levels in PCOS groups were ghrelin and PYY (24), which highlighted the importance of ghrelin and PYY in the pathogenesis of certain parameters of PCOS.

Several recent studies have demonstrated a link between the intestinal microbiota and PCOS. These studies involved women from Europe and China diagnosed with PCOS (25, 26). PCOS was associated with a change in the overall composition of the bacteria in the gut, including a decrease in alpha diversity, as well as with changes in beta diversity. In the present study, we also observed an imbalance in the intestinal microbiota in PCOS patients. Previous studies confirmed that sex hormone levels are linked to changes in the gut microbiome, and many women with PCOS have hyperandrogenism, which is associated with metabolic dysregulation (13). Accordingly, the microbial imbalance in the gut can be attributed to PCOS, and the conclusion was verified by the subsequent statistical analysis of the Adonis test results. In addition to detecting changes in the overall composition of the gut microbiome, we revealed changes in the relative abundances of specific species in women with PCOS by preforming MGS analysis. Interestingly, we noted a lack of the beneficial microbes Faecalibacterium prausnitzii and *Bifidobacterium* sp. in PCOS patients, as well as the presence of the disease-related microbes Prevotella copri and Collinsella aerofaciens. *Bifidobacterium* plays an important role in the maintenance of human health by stimulating natural immunity and contributing to the balance of the microbiota (27). Faecalibacterium prausnitzii was reported to have anti-inflammatory properties and to contribute to gut health through butyrate production. Gut microbes such as Faecalibacterium prausnitzii and *Bifidobacterium* sp. are often referred to as “good bacteria” because they exhibit health-promoting properties (28). It is proposed that an increase in the levels of these bacteria in the gut leads to the production of SCFAs that improve gut health, increasing the barrier function of the gut and reducing the translocation of bacterial endotoxins across the gut wall, where they could produce inflammation and insulin resistance (29). Accordingly, the significant depletion of Faecalibacterium prausnitzii and *Bifidobacterium* is a typical feature of intestinal microbiota disorder, and it also explains the decline in SCFA biosynthesis in PCOS patients.

In the subsequent experiment, the PCOS-related clinical indices and the intestinal microbiota of the 14 participating patients (group R and group N) exhibited an inconsistent response to the intake of the probiotic Bifidobacterium lactis V9. In combination with the results of the Bifidobacterium lactis V9 cell viability analysis, we deduced that the effective colonization of the probiotic Bifidobacterium lactis V9 in the host GI tract is crucial for its probiotic characteristics. Furthermore, understanding the principles underlying long-term bacterial colonization in humans is crucial to the success of microbiome-based therapies (28, 30). We speculated which factors influenced the effective colonization of the probiotic Bifidobacterium lactis V9 in the host GI. A previous *in vivo* study reported that under conditions of oral administration to volunteers, the probiotic Bifidobacterium longum AH1206 persisted in the guts of more than 30% of individuals (27). Those authors determined that the strain colonization was related to the low abundance of resident B. longum and the underrepresentation of specific carbohydrate utilization genes in the gut. Thus, they concluded that phylogenetic limitations and resource availability are two potential factors that control the niche colonization of introduced microbes. In the present study, we observed similar results. The relative abundance of the genus *Bifidobacterium* in the members of group R (*n* = 9) at baseline was 0.62, which was slightly lower than the relative abundance in the members of group N (0.81, *n* = 5), but the difference was not statistically significant. At the beginning of the experiment, a total of 31 PCOS patients signed the informed consent form and agreed to participate in the probiotic consumption stage without consuming other drugs. However, due to their physical and psychological factors, 17 patients began to consume a PCOS-related drug during the experiment. Therefore, we had to exclude the samples from the 17 volunteers from the remainder of the study, and only 14 patients finished the entire experiment as designed. Accordingly, the small-scale cohort limited the statistical significance of the quantification data (31). However, considering that the probiotic Bifidobacterium lactis V9 used in the present research belongs to the genus *Bifidobacterium*, we also highlighted the competitive exclusion mechanism of the same phylogenetic microbes that controls the niche colonization of introduced microbes (28).

**FIG 8:**
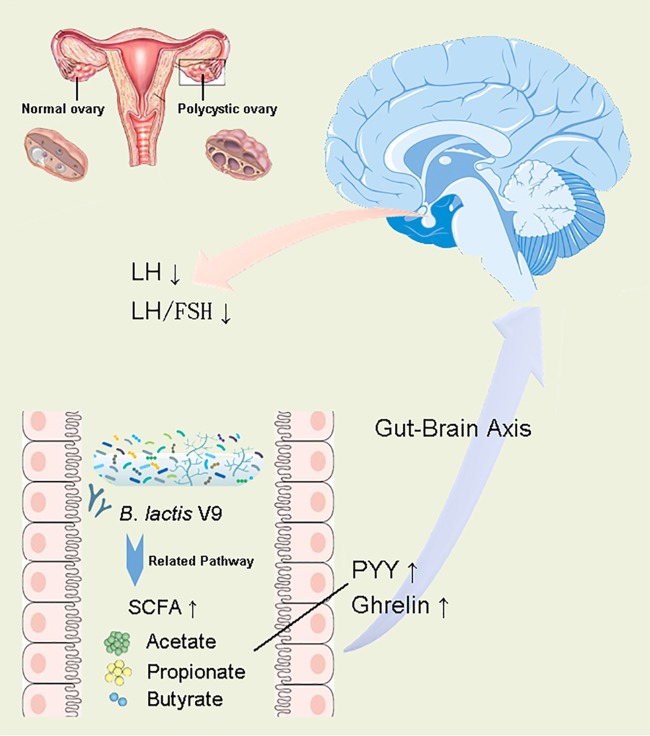
The potential mechanism by which the probiotic Bifidobacterium lactis V9 regulates the levels of sex hormones through the intestinal microbiome in PCOS patients.

Taking into account the observations of the impact of Bifidobacterium lactis V9 on the intestinal microbiota and the correlation between the intestinal microbiota and PCOS-related clinical indices and SCFA levels, we propose a potential mechanism (Fig. 8) by which the probiotic Bifidobacterium lactis V9 regulates the levels of sex hormones through the intestinal microbiome in PCOS patients. The regulatory mechanism can be described as follows. Consumption of the probiotic Bifidobacterium lactis V9 promotes the growth of SCFA-producing microbes, such as Faecalibacterium prausnitzii, *Butyricimonas*, and *Akkermansia*. These microbes, as well as *Bifidobacterium*, produce increased levels of SCFAs, impacting the secretion of gut-brain mediators, including ghrelin and PYY. Finally, changes in PYY and ghrelin levels lead to fluctuations in the levels of sex hormones secreted by the hypophysis and hypothalamus through the gut-brain axis. In this context, the probiotic Bifidobacterium lactis V9 may promote the competitive exclusion of other non-SCFA-producing bacteria by the direct inhibitory actions or competitive activity exerted by the probiotic strain or through the influence of the probiotic strain on the endogenous commensal microbiota (32–34). In line with our present study, by supplying probiotics containing *Lactobacillus*, *Bifidobacterium*, and selenium, Jamilian et al. demonstrated that probiotic and selenium coadministration in women with PCOS resulted in decreased modified Ferriman-Gallwey scores and in decreased total levels of testosterone and significantly improved mental health parameters (35). They concluded that the potential mechanisms by which probiotics can improve hormonal secretion were related to the reconstruction of the intestinal microbiome, the enhancement of digestion and absorption of dietary nutrients (36), and the interaction with the gut-brain axis (37). Communication from the intestinal microbes to the central nervous system primarily occurs through microbially derived regulators, with the best examples including SCFAs and tryptophan metabolites. Although some of these intermediates interact directly with enteroendocrine cells, enterochromaffin cells, and the mucosal immune system, resulting in propagation of “bottom-up” signaling, other intermediates are able to cross the intestinal barrier to enter the systemic circulation and may even cross the blood-brain barrier (38). Acetate, propionate, and butyrate are the primary short-chain fatty acids (SCFAs) produced by the gut bacteria from various substrates, including nondigestible fiber (39), and these SCFAs exert multiorgan effects on the host energy metabolism (29, 40). Receptors for SCFAs are present on enteroendocrine cells, and propionate can directly activate the release of PYY and GLP-1 from L-cells both *in vitro* and *in vivo* in humans (38, 41, 42). Finally, the observed negative correlation between the levels of the gut-brain axis mediators, including PYY and ghrelin, and those of LH, a typical PCOS-related sex hormone, has been widely reported (12, 31, 43, 44). In summary, the intestinal microbiota and metabolic SCFAs may play a key role in the regulation of sex and gut hormones in individuals with PCOS.

The results of the present study revealed alterations in the intestinal microbiomes of PCOS patients and identified several PCOS-related microbial biomarkers. Furthermore, we discovered a potential mechanism by which the probiotic Bifidobacterium lactis V9 modulates sex hormone levels in individuals with PCOS through the gut-brain axis. The results of this study provide new insights into the pathogenesis and treatment of PCOS and offer objective and detailed data supporting the use of probiotic treatments for metabolic diseases.

## MATERIALS AND METHODS

### Experimental design and subject recruitment.

The experimental cohort consisted of 64 subjects who were divided into two groups.

The first group (case group) consisted of 38 PCOS patients (aged 22 to 38 years) who were recruited from the Hainan General Hospital, located in Haikou, Hainan Province of China. The diagnosis of PCOS was confirmed by a B-ultrasound scanner and diagnosis of serious oligomenorrhea and by assessing the gonadal hormone levels of the individuals. The second group (control group) consisted of 26 healthy individuals (aged 23 to 30 years). After being informed of the experimental guidelines and details, 14 volunteers agreed to participate in the subsequent probiotic Bifidobacterium lactis V9 intake experiment, which was designed to evaluate the impact of Bifidobacterium lactis V9 on the intestinal microbiome of individuals with PCOS. All 14 subjects were asked to consume a total of 10.6 log CFU of Bifidobacterium lactis V9 once daily for 10 weeks. During the experiment, the subjects were asked to avoid ingesting any other probiotic products or antibiotics. Fecal samples were collected at weeks 0, 2, 4, and 10. Additionally, to evaluate the extent of Bifidobacterium lactis V9 colonization, we set up a 3-day washout period after every sampling time point, and fecal samples were also collected during the washout periods. The study was approved by the Ethical Committee of Hainan University (Haikou, China) (HNU-EC-2017011), and informed consent was obtained from all 64 volunteers before they enrolled in the study. Sampling and all described subsequent steps were conducted in accordance with the approved guidelines.

Fecal samples were collected from each individual in the morning before their first meal. After the weight of the fecal sample was determined, a sample protector (CWBIO, China) was added at a ratio of one part fecal sample to five parts of the sample protector, after which the samples were stored at −20°C until further processing. All samples were used for high-throughput sequencing of the V3-V4 region of the bacterial 16S rRNA gene, and 40 samples (including 20 from the PCOS patients and 20 from the healthy control individuals) were selected for deep shotgun metagenomic sequencing.

### DNA extraction and high-throughput sequencing of the bacterial 16S rRNA gene V3-V4 region.

The gut microbiota of each patient was isolated, and a QIAamp DNA stool minikit (Qiagen, Hilden, Germany) was used for metagenomic DNA extraction. The quality of the metagenomic DNA was assessed by spectrophotometry. We selected the bacterial V3-V4 region of the 16S rRNA gene for high-throughput sequencing (45). After PCR amplification, the amplicons were quantified, and all PCR products were subsequently pooled in equimolar ratios to reach a final concentration of 100 nmol/liter each. Next, samples were loaded onto an Illumina MiSeq high-throughput sequencing platform for sequencing (17). The QIIME (v1.7) (46) platform was used for the bioinformatics analysis. Representative operational taxonomic units (OTUs) were selected and annotated by the Ribosomal Database Project (RDP) for further microbial structure and taxonomic analyses (47).

### Deep shotgun metagenomic sequencing and quality control.

We selected 40 samples, including 20 samples from PCOS patients and 20 samples from healthy subjects, for deep shotgun metagenomic sequencing using an Illumina HiSeq 2500 instrument. Libraries were prepared with a fragment length of approximately 300 bp. Paired-end reads were generated using 100 bp in the forward and reverse directions. The reads were trimmed using Sickle and were subsequently aligned to the human genome to remove the host DNA fragments. An average of 19.17 Gb of high-quality paired-end reads, were obtained from each sample, totaling 815.32 Gb of high-quality data that were free of human DNA and adaptor contaminants (see Table S3 in the supplemental material).

### Nonredundant gene catalogue construction and calculation of gene abundance.

The shotgun reads were assembled into contigs and scaffolds using IDBA-UD (48), and then the contigs were used to predict the functional genes by the use of MetaGeneMark (49). Finally, a nonredundant gene catalogue that contained 2,382,195 genes was constructed using CD-HIT (50).

The abundances of the genes were determined by aligning the reads to the gene catalogue using Bowtie2 (51). Subsequently, for any sample N, we calculated the abundance in two steps as follows: step 1 (calculation of the copy number of each gene), $b_{i}=\frac{x_{i}}{L_{i}}$; step 2 (calculation of the relative abundance of gene *i*), $a_{i}=\frac{b_{i}}{\sum_{i}b_{i}}$ (where *a_i_* represents the relative abundance of gene *i*, *b_i_* represents the copy number of gene *i* from sample *N*, *L_i_* represents the length of gene *i*, and *x_i_* represents the number of mapped reads).

### Metagenomic species (MGS) analysis.

For the metagenomic species (MGS) analysis, the coabundance principle and canopy clustering algorithm were used to generate CAGs by binning the shotgun reads. Any CAG member that had more than 50 contigs was considered to represent an MGS. After reassembling, the MGS were assigned to a given genome when more than 80% of the subgenes matched the same genome using BLASTn at a threshold of 95% identity over 90% of the gene length. If more than 80% of the genes from an MGS had the same taxonomic level of assignment, then that MGS was identified as representing the same microbe.

### Functional annotation and metabolic pathway analysis.

The annotated amino acid sequences were aligned against the Kyoto Encyclopedia of Genes and Genomes (KEGG) databases using BLASTp (E value of ≤e−5 with a bit score higher than 60). The annotated sequences were assigned to a KEGG orthologue (KO) group according to the highest score (52). The reporter Z-scores were calculated to reveal the differences in enriched metabolic pathways between the control and PCOS groups as previously described (53). Accordingly, a reporter score of >2.3 (90% confidence according to the normal distribution) was used as a detection threshold to significantly differentiate between pathways (3).

### Quantification of Bifidobacterium lactis V9.

The Bifidobacterium lactis V9 strain-specific primer was designed according to a specific fragment (317 bp) in the complete sequence of the strains. The primer sequences were as follows: V9F, 5′-ACCCATCTCATCCAAACCAAG-3′; V9R, 5′-CACCCAAATGTAAGGAAAGTCG-3′. The specificity of the Bifidobacterium lactis V9 strain-specific primer was confirmed by PCR using DNA from other *Bifidobacterium* strains and common intestinal microbes. Each PCR mixture (20 μl) contained 10 mM Tris-HCl (pH 8.3), 50 mM KCl, 1.5 mM MgCl_2_, a 200 μM concentration of each deoxynucleoside triphosphate (dNTP), 1.5 U *Taq* DNA polymerase (TaKaRa, Japan), 0.4 μM concentrations of the primers, and 10 ng template DNA. The amplification program consisted of 1 cycle of 94°C for 2 min; 28 cycles of 94°C for 45 s, 60°C for 30 s, and 72°C for 45 s; and, finally, 1 cycle of 72°C for 7 min. PCR products were electrophoresed at 50 V in a 1.0% agarose gel. The specific band was then extracted, purified, and sequenced to confirm the specificity further.

To quantify the number of Bifidobacterium lactis V9 cells in the present study, q-PCR was performed in an ABI Step-One detection system (Applied Biosystems). To ensure that the quantified Bifidobacterium lactis V9 cells were alive, we treated the samples with propidium monoazide and phorbol myristate acetate (PMA) (30 μM; 8-min exposure time), according to the manufacturer’s instructions. The reaction mixture (20 μl) contained 10 mM Tris-HCl (pH 8.3), 50 mM KCl, 1.5 mM MgCl_2_, a 200 μM concentration of each dNTP, 0.4 μl of SYBR green I (Invitrogen), 0.4 U *Taq* DNA polymerase (Hot Start version) (TaKaRa), 0.2 μM concentrations of the specific primers, and 2 μl of template DNA. The amplification program consisted of 1 cycle of 94°C for 20 s; 40 cycles of 94°C for 5 s, 60°C for 30 s, and 72°C for 45 s; and, finally, 1 cycle of 72°C for 180 s. Fluorescence intensities were detected during the last step of each cycle. To distinguish the targeted PCR product from nontargeted PCR products, melting curves were obtained after amplification by slow heating from 58 to 95°C in increments of 0.2°C with continuous fluorescence collection. At the moment when the digital signals were captured, they were transformed into values representing the amounts of Bifidobacterium lactis V9 in each sample according to the quantified standard curve and, finally, converted into values representing the amount of Bifidobacterium lactis V9 in each gram sample.

### Measurements of metabolic parameters, sex hormones, gut-brain mediators, and intestinal SCFAs.

Blood samples were collected at the Hainan Provincial People’s Hospital for the measurements of clinical indices, including metabolic parameters (TGs, TC, and FPG), sex hormones (LH, FSH, LH/FSH, E2, PRL, and T) and gut-brain mediators (PYY and ghrelin). The sex hormones were measured on day 3 of the menstrual cycle. The blood biochemical indices were measured on an automatic biochemical analyzer (BS-220; Mindray, China), and the sex hormones were tested using an automated immunoassay system (AIA-1200; Tosoh, Japan). The ghrelin and PYY levels were determined using commercially available enzyme-linked immunosorbent assay (ELISA) kits according to the manufacturer’s instructions (12). To determine SCFA levels, we used approximately 250 mg of frozen fecal samples. The concentrations of SCFAs were determined using a 1:25 dilution of 500 μl of supernatant. Gas chromatography-mass spectrometry (GC-MS) determinations were performed using a Varian Saturn 2000 GC-MS instrument with an 8200 CX solid-phase microextraction (SPME) autosampler (54).

### Statistical analysis.

All statistical analyses were performed using R software. The pipeline used in the present study has been uploaded to the GitHub, and the link is https://github.com/zhjch321123/PCOS_Probiotics.git. The PCoA analysis was performed in R using the ade4 (55) package. The differential abundances of genera, genes, and KOs were tested with the Wilcoxon rank sum test and were considered significantly different with *P* values of *<*0.01. For pathway bubble plot construction, the package ggplot2 was used. For boxplot construction, the package ggpubr was used. The heat map was constructed using the “pheatmap” package, and the Sankey diagram was built using the “riverplot” package. The networks were calculated using the Spearman rank correlation coefficient and were visualized in Cytoscape (version 3.4).

### Data availability.

The sequence data reported in this paper have been deposited in the NCBI database (accession no. PRJNA513209 [metagenomic sequencing data]).
