# Supplementary figures and images for: Probiotic Bifidobacterium lactis V9 Regulates the Secretion of Sex Hormones in Polycystic Ovary Syndrome Patients through the Gut-Brain Axis
Source: mSystems. 2019 Apr 16;4(2):e00017-19. doi: 10.1128/mSystems.00017-19 (PMC6469956; doi:10.1128/mSystems.00017-19)

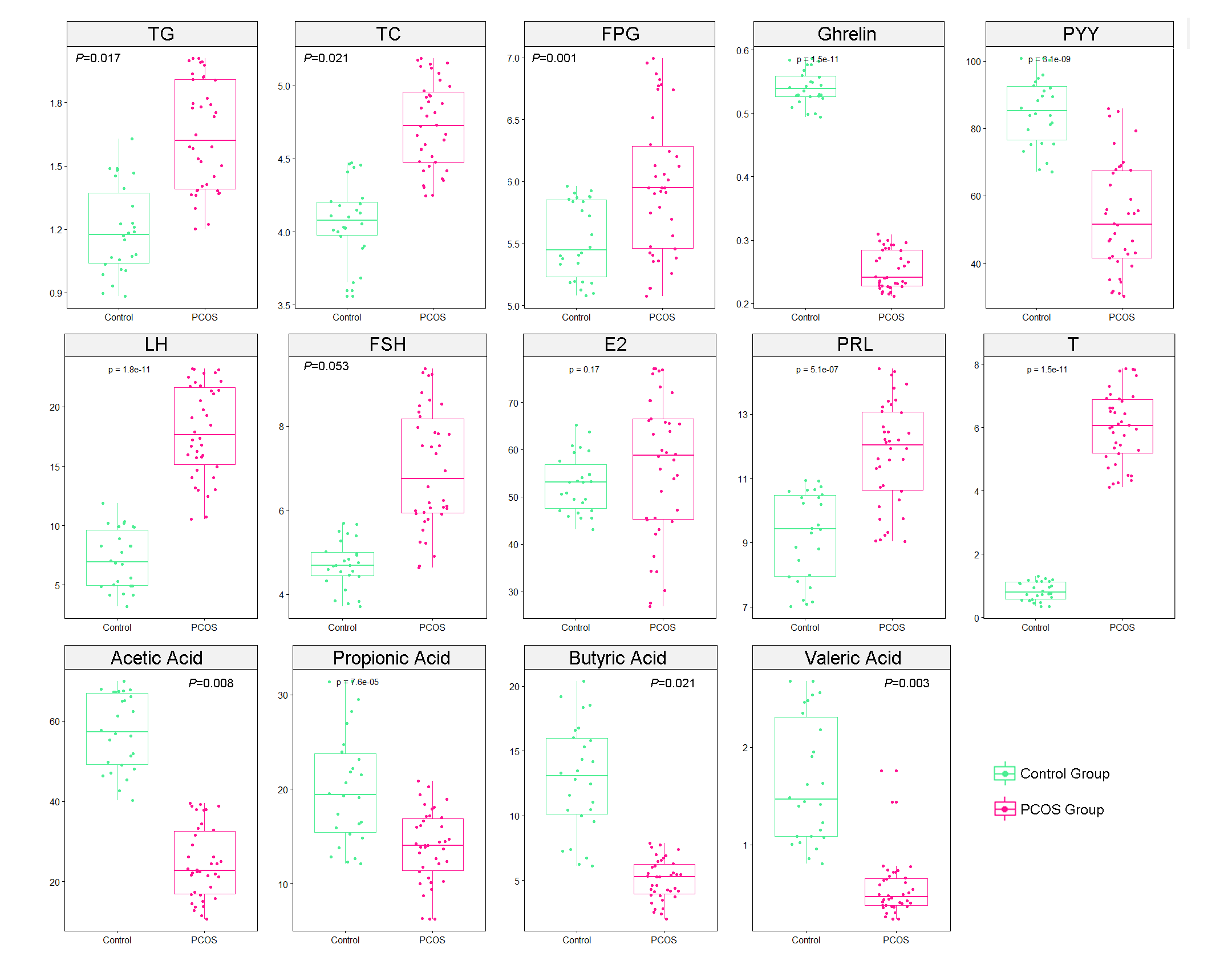

Supplement: FIG S1 [file mSystems.00017-19-sf001.tif]

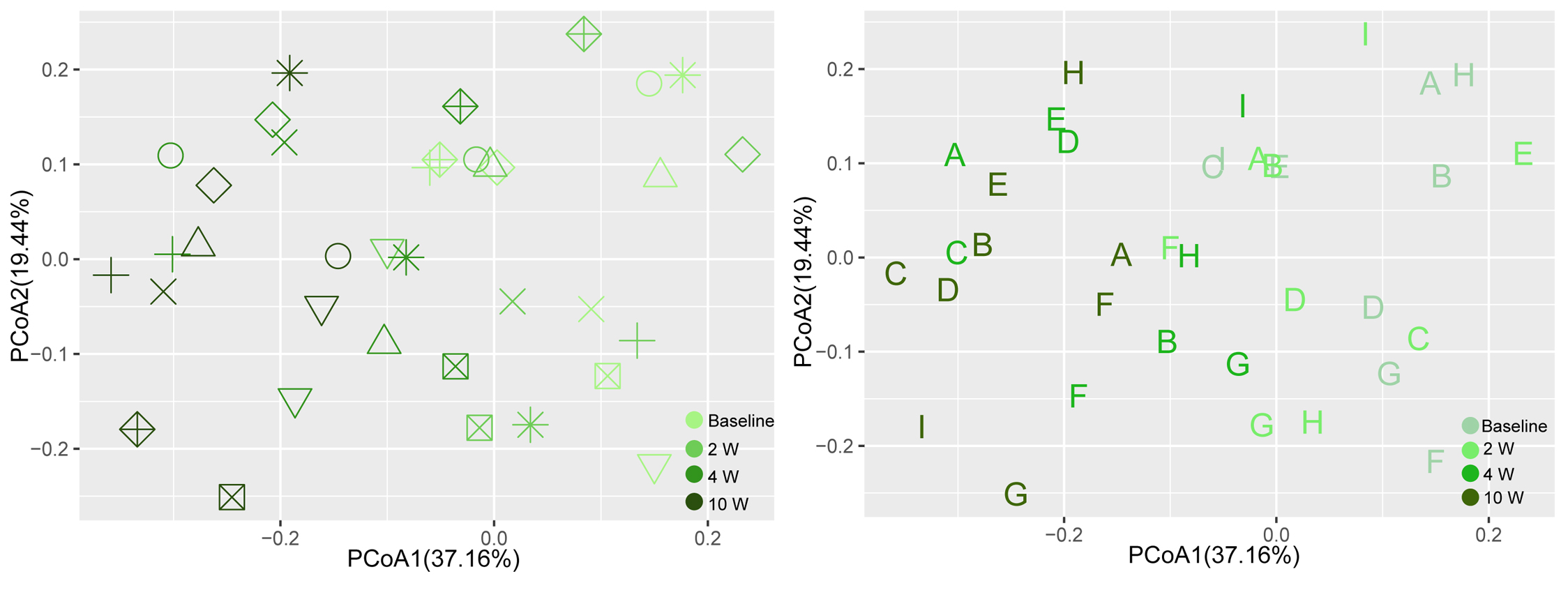

Supplement: FIG S2 [file mSystems.00017-19-sf002.tif]
