# Supplementary material for: Probiotic Bifidobacterium lactis V9 Regulates the Secretion of Sex Hormones in Polycystic Ovary Syndrome Patients through the Gut-Brain Axis
Source: mSystems. 2019 Apr 16;4(2):e00017-19. doi: 10.1128/mSystems.00017-19 (PMC6469956; doi:10.1128/mSystems.00017-19)
Supplement: TABLE S2 [file mSystems.00017-19-st002.docx]

**Table S2 Weighted and unweighted UniFrac distance based variables Adonis tests**

| #Weighted distance based variables Adonis tests | | | | | | | #UnWeighted distance based variables Adonis tests | | | | | | |
| --- | --- | --- | --- | --- | --- | --- | --- | --- | --- | --- | --- | --- | --- |
|  | Df | SumsOfSqs | MeanSqs | F.Model | R2 | Pr(>F) |  | Df | SumsOfSqs | MeanSqs | F.Model | R2 | Pr(>F) |
| AGE | 1 | 0.05493 | 0.054933 | 1.4813 | 0.02333 | 0.173 | **AGE** | 1 | 0.1645 | 0.164497 | 1.7111 | 0.02686 | 0.075 |
| Residuals | 62 | 2.29919 | 0.037084 | 0.97667 |  |  | **Residuals** | 62 | 5.9603 | 0.096134 | 0.97314 |  |  |
| Total | 63 | 2.35412 | 1 |  |  |  | **Total** | 63 | 6.1248 | 1 |  |  |  |
|  |  |  |  |  |  |  |  |  |  |  |  |  |  |
|  | Df | SumsOfSqs | MeanSqs | F.Model | R2 | Pr(>F) |  | Df | SumsOfSqs | MeanSqs | F.Model | R2 | Pr(>F) |
| HAIR | 1 | 0.05632 | 0.056323 | 1.5197 | 0.02393 | 0.194 | **HAIR** | 1 | 0.2713 | 0.27127 | 2.8733 | 0.04429 | 0.003 |
| Residuals | 62 | 2.2978 | 0.037061 | 0.97607 |  |  | **Residuals** | 62 | 5.8536 | 0.094412 | 0.95571 |  |  |
| Total | 63 | 2.35412 | 1 |  |  |  | **Total** | 63 | 6.1248 | 1 |  |  |  |
|  |  |  |  |  |  |  |  |  |  |  |  |  |  |
|  | Df | SumsOfSqs | MeanSqs | F.Model | R2 | Pr(>F) |  | Df | SumsOfSqs | MeanSqs | F.Model | R2 | Pr(>F) |
| Acne | 1 | 0.02686 | 0.026856 | 0.71547 | 0.01141 | 0.626 | **Acne** | 1 | 0.1313 | 0.13127 | 1.3579 | 0.02143 | 0.168 |
| Residuals | 62 | 2.32727 | 0.037537 | 0.98859 |  |  | **Residuals** | 62 | 5.9936 | 0.09667 | 0.97857 |  |  |
| Total | 63 | 2.35412 | 1 |  |  |  | **Total** | 63 | 6.1248 | 1 |  |  |  |
|  |  |  |  |  |  |  |  |  |  |  |  |  |  |
|  | Df | SumsOfSqs | MeanSqs | F.Model | R2 | Pr(>F) |  | Df | SumsOfSqs | MeanSqs | F.Model | R2 | Pr(>F) |
| Disease | 1 | 0.30128 | 0.30129 | 9.0994 | 0.12798 | 0.001 | **Disease** | 1 | 0.9829 | 0.98289 | 11.851 | 0.16048 | 0.001 |
| Residuals | 62 | 2.05284 | 0.03311 | 0.87202 |  |  | **Residuals** | 62 | 5.1419 | 0.08293 | 0.83952 |  |  |
| Total | 63 | 2.35412 | 1 |  |  |  | **Total** | 63 | 6.1248 | 1 |  |  |  |
|  |  |  |  |  |  |  |  |  |  |  |  |  |  |
|  | Df | SumsOfSqs | MeanSqs | F.Model | R2 | Pr(>F) |  | Df | SumsOfSqs | MeanSqs | F.Model | R2 | Pr(>F) |
| TG | 1 | 0.19675 | 0.196753 | 5.6544 | 0.08358 | 0.001 | **TG** | 1 | 0.4914 | 0.49141 | 5.4083 | 0.08023 | 0.001 |
| Residuals | 62 | 2.15737 | 0.034796 | 0.91642 |  |  | **Residuals** | 62 | 5.6334 | 0.09086 | 0.91977 |  |  |
| Total | 63 | 2.35412 | 1 |  |  |  | **Total** | 63 | 6.1248 | 1 |  |  |  |
|  |  |  |  |  |  |  |  |  |  |  |  |  |  |
|  | Df | SumsOfSqs | MeanSqs | F.Model | R2 | Pr(>F) |  | Df | SumsOfSqs | MeanSqs | F.Model | R2 | Pr(>F) |
| TC | 1 | 0.19934 | 0.199342 | 5.7357 | 0.08468 | 0.001 | **TC** | 1 | 0.6177 | 0.6177 | 6.9541 | 0.10085 | 0.001 |
| Residuals | 62 | 2.15478 | 0.034755 | 0.91532 |  |  | **Residuals** | 62 | 5.5071 | 0.08882 | 0.89915 |  |  |
| Total | 63 | 2.35412 | 1 |  |  |  | **Total** | 63 | 6.1248 | 1 |  |  |  |
|  |  |  |  |  |  |  |  |  |  |  |  |  |  |
|  | Df | SumsOfSqs | MeanSqs | F.Model | R2 | Pr(>F) |  | Df | SumsOfSqs | MeanSqs | F.Model | R2 | Pr(>F) |
| FPG | 1 | 0.05141 | 0.05141 | 1.3842 | 0.02184 | 0.211 | **FPG** | 1 | 0.2516 | 0.25159 | 2.6558 | 0.04108 | 0.006 |
| Residuals | 62 | 2.30271 | 0.037141 | 0.97816 |  |  | **Residuals** | 62 | 5.8733 | 0.09473 | 0.95892 |  |  |
| Total | 63 | 2.35412 | 1 |  |  |  | **Total** | 63 | 6.1248 | 1 |  |  |  |
|  |  |  |  |  |  |  |  |  |  |  |  |  |  |
|  | Df | SumsOfSqs | MeanSqs | F.Model | R2 | Pr(>F) |  | Df | SumsOfSqs | MeanSqs | F.Model | R2 | Pr(>F) |
| LH | 1 | 0.24293 | 0.242931 | 7.1342 | 0.10319 | 0.001 | **LH** | 1 | 0.7897 | 0.78974 | 9.1777 | 0.12894 | 0.001 |
| Residuals | 62 | 2.11119 | 0.034051 | 0.89681 |  |  | **Residuals** | 62 | 5.3351 | 0.08605 | 0.87106 |  |  |
| Total | 63 | 2.35412 | 1 |  |  |  | **Total** | 63 | 6.1248 | 1 |  |  |  |
|  |  |  |  |  |  |  |  |  |  |  |  |  |  |
|  | Df | SumsOfSqs | MeanSqs | F.Model | R2 | Pr(>F) |  | Df | SumsOfSqs | MeanSqs | F.Model | R2 | Pr(>F) |
| FSH | 1 | 0.15768 | 0.157681 | 4.4509 | 0.06698 | 0.005 | **FSH** | 1 | 0.526 | 0.52601 | 5.8248 | 0.08588 | 0.001 |
| Residuals | 62 | 2.19644 | 0.035426 | 0.93302 |  |  | **Residuals** | 62 | 5.5988 | 0.0903 | 0.91412 |  |  |
| Total | 63 | 2.35412 | 1 |  |  |  | **Total** | 63 | 6.1248 | 1 |  |  |  |
|  |  |  |  |  |  |  |  |  |  |  |  |  |  |
|  | Df | SumsOfSqs | MeanSqs | F.Model | R2 | Pr(>F) |  | Df | SumsOfSqs | MeanSqs | F.Model | R2 | Pr(>F) |
| E2 | 1 | 0.0205 | 0.020496 | 0.54454 | 0.00871 | 0.74 | **E2** | 1 | 0.1201 | 0.12011 | 1.2402 | 0.01961 | 0.253 |
| Residuals | 62 | 2.3336 | 0.037639 | 0.99129 |  |  | **Residuals** | 62 | 6.0047 | 0.09685 | 0.98039 |  |  |
| Total | 63 | 2.3541 | 1 |  |  |  | **Total** | 63 | 6.1248 | 1 |  |  |  |
|  |  |  |  |  |  |  |  |  |  |  |  |  |  |
|  | Df | SumsOfSqs | MeanSqs | F.Model | R2 | Pr(>F) |  | Df | SumsOfSqs | MeanSqs | F.Model | R2 | Pr(>F) |
| PRL | 1 | 0.15388 | 0.153884 | 4.3363 | 0.06537 | 0.002 | **PRL** | 1 | 0.4768 | 0.47676 | 5.2335 | 0.07784 | 0.001 |
| Residuals | 62 | 2.20024 | 0.035488 | 0.93463 |  |  | **Residuals** | 62 | 5.6481 | 0.0911 | 0.92216 |  |  |
| Total | 63 | 2.35412 | 1 |  |  |  | **Total** | 63 | 6.1248 | 1 |  |  |  |
|  |  |  |  |  |  |  |  |  |  |  |  |  |  |
|  | Df | SumsOfSqs | MeanSqs | F.Model | R2 | Pr(>F) |  | Df | SumsOfSqs | MeanSqs | F.Model | R2 | Pr(>F) |
| T | 1 | 0.24707 | 0.247068 | 7.27 | 0.10495 | 0.001 | **T** | 1 | 0.8597 | 0.85966 | 10.123 | 0.14036 | 0.001 |
| Residuals | 62 | 2.10705 | 0.033985 | 0.89505 |  |  | **Residuals** | 62 | 5.2652 | 0.08492 | 0.85964 |  |  |
| Total | 63 | 2.35412 | 1 |  |  |  | **Total** | 63 | 6.1248 | 1 |  |  |  |
|  |  |  |  |  |  |  |  |  |  |  |  |  |  |
|  | Df | SumsOfSqs | MeanSqs | F.Model | R2 | Pr(>F) |  | Df | SumsOfSqs | MeanSqs | F.Model | R2 | Pr(>F) |
| Ghrelin | 1 | 0.29856 | 0.298564 | 9.0053 | 0.12683 | 0.001 | **Ghrelin** | 1 | 0.9223 | 0.92229 | 10.991 | 0.15058 | 0.001 |
| Residuals | 62 | 2.05556 | 0.033154 | 0.87317 |  |  | **Residuals** | 62 | 5.2025 | 0.08391 | 0.84942 |  |  |
| Total | 63 | 2.35412 | 1 |  |  |  | **Total** | 63 | 6.1248 | 1 |  |  |  |
|  |  |  |  |  |  |  |  |  |  |  |  |  |  |
|  | Df | SumsOfSqs | MeanSqs | F.Model | R2 | Pr(>F) |  | Df | SumsOfSqs | MeanSqs | F.Model | R2 | Pr(>F) |
| PYY | 1 | 0.19249 | 0.192485 | 5.5209 | 0.08177 | 0.001 | **PYY** | 1 | 0.5392 | 0.5392 | 5.9851 | 0.08804 | 0.001 |
| Residuals | 62 | 2.16164 | 0.034865 | 0.91823 |  |  | **Residuals** | 62 | 5.5856 | 0.09009 | 0.91196 |  |  |
| Total | 63 | 2.35412 | 1 |  |  |  | **Total** | 63 | 6.1248 | 1 |  |  |  |
|  |  |  |  |  |  |  |  |  |  |  |  |  |  |
|  | Df | SumsOfSqs | MeanSqs | F.Model | R2 | Pr(>F) |  | Df | SumsOfSqs | MeanSqs | F.Model | R2 | Pr(>F) |
| Acetic.acid | 1 | 0.25461 | 0.254606 | 7.5187 | 0.10815 | 0.001 | **Acetic.acid** | 1 | 0.7295 | 0.72953 | 8.3834 | 0.11911 | 0.001 |
| Residuals | 62 | 2.09952 | 0.033863 | 0.89185 |  |  | **Residuals** | 62 | 5.3953 | 0.08702 | 0.88089 |  |  |
| Total | 63 | 2.35412 | 1 |  |  |  | **Total** | 63 | 6.1248 | 1 |  |  |  |
|  |  |  |  |  |  |  |  |  |  |  |  |  |  |
|  | Df | SumsOfSqs | MeanSqs | F.Model | R2 | Pr(>F) |  | Df | SumsOfSqs | MeanSqs | F.Model | R2 | Pr(>F) |
| Propionic.acid | 1 | 0.12231 | 0.122309 | 3.3978 | 0.05196 | 0.007 | **Propionic.acid** | 1 | 0.3107 | 0.31066 | 3.3128 | 0.05072 | 0.001 |
| Residuals | 62 | 2.23181 | 0.035997 | 0.94804 |  |  | **Residuals** | 62 | 5.8142 | 0.093777 | 0.94928 |  |  |
| Total | 63 | 2.35412 | 1 |  |  |  | **Total** | 63 | 6.1248 | 1 |  |  |  |
|  |  |  |  |  |  |  |  |  |  |  |  |  |  |
|  | Df | SumsOfSqs | MeanSqs | F.Model | R2 | Pr(>F) |  | Df | SumsOfSqs | MeanSqs | F.Model | R2 | Pr(>F) |
| Butyric.acid | 1 | 0.21882 | 0.21882 | 6.3535 | 0.09295 | 0.002 | **Butyric.acid** | 1 | 0.6187 | 0.61871 | 6.9668 | 0.10102 | 0.001 |
| Residuals | 62 | 2.13531 | 0.03444 | 0.90705 |  |  | **Residuals** | 62 | 5.5061 | 0.08881 | 0.89898 |  |  |
| Total | 63 | 2.35412 | 1 |  |  |  | **Total** | 63 | 6.1248 | 1 |  |  |  |
|  |  |  |  |  |  |  |  |  |  |  |  |  |  |
|  | Df | SumsOfSqs | MeanSqs | F.Model | R2 | Pr(>F) |  | Df | SumsOfSqs | MeanSqs | F.Model | R2 | Pr(>F) |
| Valeric.acid | 1 | 0.20217 | 0.202166 | 5.8246 | 0.08588 | 0.001 | **Valeric.acid** | 1 | 0.5937 | 0.59366 | 6.6545 | 0.09693 | 0.001 |
| Residuals | 62 | 2.15196 | 0.034709 | 0.91412 |  |  | **Residuals** | 62 | 5.5312 | 0.08921 | 0.90307 |  |  |
| Total | 63 | 2.35412 | 1 |  |  |  | **Total** | 63 | 6.1248 | 1 |  |  |  |
