# Supplementary material for: Probiotic Bifidobacterium lactis V9 Regulates the Secretion of Sex Hormones in Polycystic Ovary Syndrome Patients through the Gut-Brain Axis
Source: mSystems. 2019 Apr 16;4(2):e00017-19. doi: 10.1128/mSystems.00017-19 (PMC6469956; doi:10.1128/mSystems.00017-19)
Supplement: TABLE S3 [file mSystems.00017-19-st003.docx]

**Table S3 Weighted and unweighted UniFrac distance based metagenomic species Adonis tests**

| #Weighted distance based metagenomic species Adonis tests | | | | | | | #UnWeighted distance based metagenomic species Adonis tests | | | | |
| --- | --- | --- | --- | --- | --- | --- | --- | --- | --- | --- | --- |
|  | Df | SumsOfSqs | MeanSqs | F.Model | R2 | Pr(>F) | SumsOfSqs | MeanSqs | F.Model | R2 | Pr(>F) |
| Alistipes.putredinis.MGS041 | 1 | 0.07253 | 0.072531 | 1.8918 | 0.09511 | 0.102 | 0.21092 | 0.210916 | 2.3979 | 0.11756 | 0.019 |
| Residuals | 38 | 0.6901 | 0.038339 | 0.90489 |  |  | 1.58324 | 0.087958 | 0.88244 |  |  |
| Total | 39 | 0.76264 | 1 |  |  |  | 1.79415 | 1 |  |  |  |
|  | Df | SumsOfSqs | MeanSqs | F.Model | R2 | Pr(>F) | SumsOfSqs | MeanSqs | F.Model | R2 | Pr(>F) |
| Bacteroides.caccae.MGS005 | 1 | 0.0474 | 0.047405 | 1.193 | 0.06216 | 0.271 | 0.0981 | 0.098098 | 1.0411 | 0.05468 | 0.455 |
| Residuals | 38 | 0.71523 | 0.039735 | 0.93784 |  |  | 1.6961 | 0.094225 | 0.94532 |  |  |
| Total | 39 | 0.76264 | 1 |  |  |  | 1.7941 | 1 |  |  |  |
|  | Df | SumsOfSqs | MeanSqs | F.Model | R2 | Pr(>F) | SumsOfSqs | MeanSqs | F.Model | R2 | Pr(>F) |
| Bacteroides.caccae.MGS018 | 1 | 0.0326 | 0.0326 | 0.80381 | 0.04275 | 0.519 | 0.16215 | 0.162149 | 1.7884 | 0.09038 | 0.07 |
| Residuals | 38 | 0.73004 | 0.040558 | 0.95725 |  |  | 1.632 | 0.090667 | 0.90962 |  |  |
| Total | 39 | 0.76264 | 1 |  |  |  | 1.79415 | 1 |  |  |  |
|  | Df | SumsOfSqs | MeanSqs | F.Model | R2 | Pr(>F) | SumsOfSqs | MeanSqs | F.Model | R2 | Pr(>F) |
| Bacteroides.plebeius.MGS006 | 1 | 0.06608 | 0.066084 | 1.7077 | 0.08665 | 0.131 | 0.18059 | 0.180593 | 2.0146 | 0.10066 | 0.017 |
| Residuals | 38 | 0.69655 | 0.038697 | 0.91335 |  |  | 1.61356 | 0.089642 | 0.89934 |  |  |
| Total | 39 | 0.76264 | 1 |  |  |  | 1.79415 | 1 |  |  |  |
|  | Df | SumsOfSqs | MeanSqs | F.Model | R2 | Pr(>F) | SumsOfSqs | MeanSqs | F.Model | R2 | Pr(>F) |
| Bacteroides.plebeius.MGS058 | 1 | 0.0615 | 0.061502 | 1.5789 | 0.08064 | 0.147 | 0.09212 | 0.092122 | 0.97424 | 0.05135 | 0.454 |
| Residuals | 38 | 0.70113 | 0.038952 | 0.91936 |  |  | 1.70203 | 0.094557 | 0.94865 |  |  |
| Total | 39 | 0.76264 | 1 |  |  |  | 1.79415 | 1 |  |  |  |
|  | Df | SumsOfSqs | MeanSqs | F.Model | R2 | Pr(>F) | SumsOfSqs | MeanSqs | F.Model | R2 | Pr(>F) |
| Bacteroides.sp..MGS001 | 1 | 0.01391 | 0.013908 | 0.33436 | 0.01824 | 0.899 | 0.07477 | 0.074768 | 0.78273 | 0.04167 | 0.608 |
| Residuals | 38 | 0.74873 | 0.041596 | 0.98176 |  |  | 1.71938 | 0.095521 | 0.95833 |  |  |
| Total | 39 | 0.76264 | 1 |  |  |  | 1.79415 | 1 |  |  |  |
|  | Df | SumsOfSqs | MeanSqs | F.Model | R2 | Pr(>F) | SumsOfSqs | MeanSqs | F.Model | R2 | Pr(>F) |
| Bacteroides.sp..MGS008 | 1 | 0.05034 | 0.05034 | 1.2721 | 0.06601 | 0.263 | 0.17074 | 0.17074 | 1.8931 | 0.09516 | 0.034 |
| Residuals | 38 | 0.7123 | 0.039572 | 0.93399 |  |  | 1.62341 | 0.09019 | 0.90484 |  |  |
| Total | 39 | 0.76264 | 1 |  |  |  | 1.79415 | 1 |  |  |  |
|  | Df | SumsOfSqs | MeanSqs | F.Model | R2 | Pr(>F) | SumsOfSqs | MeanSqs | F.Model | R2 | Pr(>F) |
| Bacteroides.sp..MGS019 | 1 | 0.08697 | 0.086966 | 2.3168 | 0.11403 | 0.066 | 0.11532 | 0.115323 | 1.2365 | 0.06428 | 0.265 |
| Residuals | 38 | 0.67567 | 0.037537 | 0.88597 |  |  | 1.67883 | 0.093268 | 0.93572 |  |  |
| Total | 39 | 0.76264 | 1 |  |  |  | 1.79415 | 1 |  |  |  |
|  | Df | SumsOfSqs | MeanSqs | F.Model | R2 | Pr(>F) | SumsOfSqs | MeanSqs | F.Model | R2 | Pr(>F) |
| Bacteroides.sp..MGS027 | 1 | 0.08873 | 0.08873 | 2.37 | 0.11635 | 0.061 | 0.30854 | 0.308537 | 3.7383 | 0.17197 | 0.001 |
| Residuals | 38 | 0.67391 | 0.037439 | 0.88365 |  |  | 1.48562 | 0.082534 | 0.82803 |  |  |
| Total | 39 | 0.76264 | 1 |  |  |  | 1.79415 | 1 |  |  |  |
|  | Df | SumsOfSqs | MeanSqs | F.Model | R2 | Pr(>F) | SumsOfSqs | MeanSqs | F.Model | R2 | Pr(>F) |
| Bacteroides.stercoris.MGS016 | 1 | 0.07633 | 0.076333 | 2.002 | 0.10009 | 0.081 | 0.12192 | 0.121918 | 1.3123 | 0.06795 | 0.27 |
| Residuals | 38 | 0.6863 | 0.038128 | 0.89991 |  |  | 1.67223 | 0.092902 | 0.93205 |  |  |
| Total | 39 | 0.76264 | 1 |  |  |  | 1.79415 | 1 |  |  |  |
|  | Df | SumsOfSqs | MeanSqs | F.Model | R2 | Pr(>F) | SumsOfSqs | MeanSqs | F.Model | R2 | Pr(>F) |
| Bacteroides.uniformis.MGS022 | 1 | 0.04624 | 0.046242 | 1.1619 | 0.06063 | 0.307 | 0.19114 | 0.191136 | 2.1462 | 0.10653 | 0.029 |
| Residuals | 38 | 0.71639 | 0.0398 | 0.93937 |  |  | 1.60302 | 0.089056 | 0.89347 |  |  |
| Total | 39 | 0.76264 | 1 |  |  |  | 1.79415 | 1 |  |  |  |
|  | Df | SumsOfSqs | MeanSqs | F.Model | R2 | Pr(>F) | SumsOfSqs | MeanSqs | F.Model | R2 | Pr(>F) |
| Bacteroides.vulgatus.MGS014 | 1 | 0.05461 | 0.05461 | 1.3883 | 0.07161 | 0.249 | 0.20615 | 0.206155 | 2.3368 | 0.1149 | 0.014 |
| Residuals | 38 | 0.70803 | 0.039335 | 0.92839 |  |  | 1.588 | 0.088222 | 0.8851 |  |  |
| Total | 39 | 0.76264 | 1 |  |  |  | 1.79415 | 1 |  |  |  |
|  | Df | SumsOfSqs | MeanSqs | F.Model | R2 | Pr(>F) | SumsOfSqs | MeanSqs | F.Model | R2 | Pr(>F) |
| Bifidobacterium.animalis.MGS015 | 1 | 0.10962 | 0.109616 | 3.0215 | 0.14373 | 0.011 | 0.32999 | 0.32999 | 4.0567 | 0.18392 | 0.001 |
| Residuals | 38 | 0.65302 | 0.036279 | 0.85627 |  |  | 1.46417 | 0.08134 | 0.81608 |  |  |
| Total | 39 | 0.76264 | 1 |  |  |  | 1.79415 | 1 |  |  |  |
|  | Df | SumsOfSqs | MeanSqs | F.Model | R2 | Pr(>F) | SumsOfSqs | MeanSqs | F.Model | R2 | Pr(>F) |
| Bifidobacterium.pseudocatenulatum.MGS052 | 1 | 0.01974 | 0.019736 | 0.47819 | 0.02588 | 0.824 | 0.10013 | 0.100128 | 1.0639 | 0.05581 | 0.44 |
| Residuals | 38 | 0.7429 | 0.041272 | 0.97412 |  |  | 1.69402 | 0.094112 | 0.94419 |  |  |
| Total | 39 | 0.76264 | 1 |  |  |  | 1.79415 | 1 |  |  |  |
|  | Df | SumsOfSqs | MeanSqs | F.Model | R2 | Pr(>F) | SumsOfSqs | MeanSqs | F.Model | R2 | Pr(>F) |
| Bifidobacterium.sp..MGS003 | 1 | 0.09119 | 0.091191 | 2.4446 | 0.11957 | 0.07 | 0.34266 | 0.34266 | 4.2494 | 0.19099 | 0.001 |
| Residuals | 38 | 0.67145 | 0.037303 | 0.88043 |  |  | 1.45149 | 0.08064 | 0.80901 |  |  |
| Total | 39 | 0.76264 | 1 |  |  |  | 1.79415 | 1 |  |  |  |
|  | Df | SumsOfSqs | MeanSqs | F.Model | R2 | Pr(>F) | SumsOfSqs | MeanSqs | F.Model | R2 | Pr(>F) |
| Bifidobacterium.sp..MGS009 | 1 | 0.09247 | 0.092474 | 2.4838 | 0.12126 | 0.039 | 0.31211 | 0.312106 | 3.7906 | 0.17396 | 0.001 |
| Residuals | 38 | 0.67016 | 0.037231 | 0.87874 |  |  | 1.48205 | 0.082336 | 0.82604 |  |  |
| Total | 39 | 0.76264 | 1 |  |  |  | 1.79415 | 1 |  |  |  |
|  | Df | SumsOfSqs | MeanSqs | F.Model | R2 | Pr(>F) | SumsOfSqs | MeanSqs | F.Model | R2 | Pr(>F) |
| Clostridium.sp..MGS002 | 1 | 0.05062 | 0.050623 | 1.2798 | 0.06638 | 0.256 | 0.10693 | 0.106926 | 1.1407 | 0.0596 | 0.345 |
| Residuals | 38 | 0.71201 | 0.039556 | 0.93362 |  |  | 1.68723 | 0.093735 | 0.9404 |  |  |
| Total | 39 | 0.76264 | 1 |  |  |  | 1.79415 | 1 |  |  |  |
|  | Df | SumsOfSqs | MeanSqs | F.Model | R2 | Pr(>F) | SumsOfSqs | MeanSqs | F.Model | R2 | Pr(>F) |
| Collinsella.aerofaciens.MGS060 | 1 | 0.07105 | 0.071051 | 1.8493 | 0.09316 | 0.116 | 0.21167 | 0.211666 | 2.4076 | 0.11798 | 0.014 |
| Residuals | 38 | 0.69158 | 0.038421 | 0.90684 |  |  | 1.58249 | 0.087916 | 0.88202 |  |  |
| Total | 39 | 0.76264 | 1 |  |  |  | 1.79415 | 1 |  |  |  |
|  | Df | SumsOfSqs | MeanSqs | F.Model | R2 | Pr(>F) | SumsOfSqs | MeanSqs | F.Model | R2 | Pr(>F) |
| Coprococcus.comes.MGS023 | 1 | 0.06674 | 0.066738 | 1.7262 | 0.08751 | 0.129 | 0.20556 | 0.205562 | 2.3292 | 0.11457 | 0.014 |
| Residuals | 38 | 0.6959 | 0.038661 | 0.91249 |  |  | 1.58859 | 0.088255 | 0.88543 |  |  |
| Total | 39 | 0.76264 | 1 |  |  |  | 1.79415 | 1 |  |  |  |
|  | Df | SumsOfSqs | MeanSqs | F.Model | R2 | Pr(>F) | SumsOfSqs | MeanSqs | F.Model | R2 | Pr(>F) |
| Coprococcus.eutactus.MGS007 | 1 | 0.065 | 0.064999 | 1.6771 | 0.08523 | 0.133 | 0.16483 | 0.164834 | 1.821 | 0.09187 | 0.029 |
| Residuals | 38 | 0.69764 | 0.038758 | 0.91477 |  |  | 1.62932 | 0.090518 | 0.90813 |  |  |
| Total | 39 | 0.76264 | 1 |  |  |  | 1.79415 | 1 |  |  |  |
|  | Df | SumsOfSqs | MeanSqs | F.Model | R2 | Pr(>F) | SumsOfSqs | MeanSqs | F.Model | R2 | Pr(>F) |
| Dialister.invisus.MGS048 | 1 | 0.01982 | 0.019821 | 0.48031 | 0.02599 | 0.829 | 0.1273 | 0.127302 | 1.3747 | 0.07095 | 0.194 |
| Residuals | 38 | 0.74281 | 0.041267 | 0.97401 |  |  | 1.6668 | 0.092603 | 0.92905 |  |  |
| Total | 39 | 0.76264 | 1 |  |  |  | 1.7941 | 1 |  |  |  |
|  | Df | SumsOfSqs | MeanSqs | F.Model | R2 | Pr(>F) | SumsOfSqs | MeanSqs | F.Model | R2 | Pr(>F) |
| Eubacterium.eligens.MGS013 | 1 | 0.06971 | 0.069708 | 1.8108 | 0.0914 | 0.109 | 0.22263 | 0.222631 | 2.55 | 0.12409 | 0.001 |
| Residuals | 38 | 0.69293 | 0.038496 | 0.9086 |  |  | 1.57152 | 0.087307 | 0.87591 |  |  |
| Total | 39 | 0.76264 | 1 |  |  |  | 1.79415 | 1 |  |  |  |
|  | Df | SumsOfSqs | MeanSqs | F.Model | R2 | Pr(>F) | SumsOfSqs | MeanSqs | F.Model | R2 | Pr(>F) |
| Eubacterium.rectale.MGS044 | 1 | 0.06755 | 0.067549 | 1.7493 | 0.08857 | 0.125 | 0.27252 | 0.272521 | 3.2238 | 0.15189 | 0.001 |
| Residuals | 38 | 0.69509 | 0.038616 | 0.91143 |  |  | 1.52163 | 0.084535 | 0.84811 |  |  |
| Total | 39 | 0.76264 | 1 |  |  |  | 1.79415 | 1 |  |  |  |
|  | Df | SumsOfSqs | MeanSqs | F.Model | R2 | Pr(>F) | SumsOfSqs | MeanSqs | F.Model | R2 | Pr(>F) |
| Faecalibacterium.prausnitzii.MGS004 | 1 | 0.03548 | 0.035478 | 0.87821 | 0.04652 | 0.448 | 0.14088 | 0.140884 | 1.5339 | 0.07852 | 0.088 |
| Residuals | 38 | 0.72716 | 0.040398 | 0.95348 |  |  | 1.65327 | 0.091848 | 0.92148 |  |  |
| Total | 39 | 0.76264 | 1 |  |  |  | 1.79415 | 1 |  |  |  |
|  | Df | SumsOfSqs | MeanSqs | F.Model | R2 | Pr(>F) | SumsOfSqs | MeanSqs | F.Model | R2 | Pr(>F) |
| Faecalibacterium.prausnitzii.MGS017 | 1 | 0.03851 | 0.038506 | 0.95716 | 0.05049 | 0.407 | 0.08797 | 0.087974 | 0.92811 | 0.04903 | 0.517 |
| Residuals | 38 | 0.72413 | 0.040229 | 0.94951 |  |  | 1.70618 | 0.094788 | 0.95097 |  |  |
| Total | 39 | 0.76264 | 1 |  |  |  | 1.79415 | 1 |  |  |  |
|  | Df | SumsOfSqs | MeanSqs | F.Model | R2 | Pr(>F) | SumsOfSqs | MeanSqs | F.Model | R2 | Pr(>F) |
| Faecalibacterium.prausnitzii.MGS021 | 1 | 0.06027 | 0.060272 | 1.5446 | 0.07903 | 0.176 | 0.16373 | 0.16373 | 1.8076 | 0.09126 | 0.053 |
| Residuals | 38 | 0.70236 | 0.03902 | 0.92097 |  |  | 1.63042 | 0.090579 | 0.90874 |  |  |
| Total | 39 | 0.76264 | 1 |  |  |  | 1.79415 | 1 |  |  |  |
|  | Df | SumsOfSqs | MeanSqs | F.Model | R2 | Pr(>F) | SumsOfSqs | MeanSqs | F.Model | R2 | Pr(>F) |
| Faecalibacterium.prausnitzii.MGS032 | 1 | 0.02476 | 0.02476 | 0.604 | 0.03247 | 0.715 | 0.15318 | 0.153177 | 1.6802 | 0.08538 | 0.094 |
| Residuals | 38 | 0.73788 | 0.040993 | 0.96753 |  |  | 1.64097 | 0.091165 | 0.91462 |  |  |
| Total | 39 | 0.76264 | 1 |  |  |  | 1.79415 | 1 |  |  |  |
|  | Df | SumsOfSqs | MeanSqs | F.Model | R2 | Pr(>F) | SumsOfSqs | MeanSqs | F.Model | R2 | Pr(>F) |
| Faecalibacterium.prausnitzii.MGS033 | 1 | 0.03346 | 0.033456 | 0.82586 | 0.04387 | 0.543 | 0.10165 | 0.101646 | 1.081 | 0.05665 | 0.392 |
| Residuals | 38 | 0.72918 | 0.04051 | 0.95613 |  |  | 1.69251 | 0.094028 | 0.94335 |  |  |
| Total | 39 | 0.76264 | 1 |  |  |  | 1.79415 | 1 |  |  |  |
|  | Df | SumsOfSqs | MeanSqs | F.Model | R2 | Pr(>F) | SumsOfSqs | MeanSqs | F.Model | R2 | Pr(>F) |
| Faecalibacterium.prausnitzii.MGS039 | 1 | 0.08308 | 0.083083 | 2.2007 | 0.10894 | 0.053 | 0.12568 | 0.125684 | 1.3559 | 0.07005 | 0.182 |
| Residuals | 38 | 0.67955 | 0.037753 | 0.89106 |  |  | 1.66847 | 0.092693 | 0.92995 |  |  |
| Total | 39 | 0.76264 | 1 |  |  |  | 1.79415 | 1 |  |  |  |
|  | Df | SumsOfSqs | MeanSqs | F.Model | R2 | Pr(>F) | SumsOfSqs | MeanSqs | F.Model | R2 | Pr(>F) |
| Faecalibacterium.prausnitzii.MGS040 | 1 | 0.03581 | 0.035814 | 0.88695 | 0.04696 | 0.458 | 0.10527 | 0.105272 | 1.122 | 0.05867 | 0.325 |
| Residuals | 38 | 0.72682 | 0.040379 | 0.95304 |  |  | 1.68888 | 0.093827 | 0.94133 |  |  |
| Total | 39 | 0.76264 | 1 |  |  |  | 1.79415 | 1 |  |  |  |
|  | Df | SumsOfSqs | MeanSqs | F.Model | R2 | Pr(>F) | SumsOfSqs | MeanSqs | F.Model | R2 | Pr(>F) |
| Faecalibacterium.prausnitzii.MGS049 | 1 | 0.01691 | 0.016913 | 0.40824 | 0.02218 | 0.854 | 0.14993 | 0.149931 | 1.6414 | 0.08357 | 0.094 |
| Residuals | 38 | 0.74572 | 0.041429 | 0.97782 |  |  | 1.64422 | 0.091346 | 0.91643 |  |  |
| Total | 39 | 0.76264 | 1 |  |  |  | 1.79415 | 1 |  |  |  |
|  | Df | SumsOfSqs | MeanSqs | F.Model | R2 | Pr(>F) | SumsOfSqs | MeanSqs | F.Model | R2 | Pr(>F) |
| Faecalibacterium.prausnitzii.MGS057 | 1 | 0.11109 | 0.111085 | 3.0689 | 0.14566 | 0.018 | 0.12322 | 0.12322 | 1.3274 | 0.06868 | 0.224 |
| Residuals | 38 | 0.65155 | 0.036197 | 0.85434 |  |  | 1.67093 | 0.09283 | 0.93132 |  |  |
| Total | 39 | 0.76264 | 1 |  |  |  | 1.79415 | 1 |  |  |  |
|  | Df | SumsOfSqs | MeanSqs | F.Model | R2 | Pr(>F) | SumsOfSqs | MeanSqs | F.Model | R2 | Pr(>F) |
| Prevotella.copri.MGS012 | 1 | 0.03145 | 0.031448 | 0.77416 | 0.04124 | 0.579 | 0.06212 | 0.062123 | 0.6456 | 0.03462 | 0.788 |
| Residuals | 38 | 0.73119 | 0.040622 | 0.95876 |  |  | 1.73203 | 0.096224 | 0.96538 |  |  |
| Total | 39 | 0.76264 | 1 |  |  |  | 1.79415 | 1 |  |  |  |
|  | Df | SumsOfSqs | MeanSqs | F.Model | R2 | Pr(>F) | SumsOfSqs | MeanSqs | F.Model | R2 | Pr(>F) |
| Prevotella.copri.MGS030 | 1 | 0.02798 | 0.027976 | 0.68544 | 0.03668 | 0.611 | 0.10162 | 0.101623 | 1.0808 | 0.05664 | 0.395 |
| Residuals | 38 | 0.73466 | 0.040814 | 0.96332 |  |  | 1.69253 | 0.094029 | 0.94336 |  |  |
| Total | 39 | 0.76264 | 1 |  |  |  | 1.79415 | 1 |  |  |  |
|  | Df | SumsOfSqs | MeanSqs | F.Model | R2 | Pr(>F) | SumsOfSqs | MeanSqs | F.Model | R2 | Pr(>F) |
| Prevotella.copri.MGS035 | 1 | 0.05776 | 0.057759 | 1.4749 | 0.07574 | 0.165 | 0.09214 | 0.092139 | 0.97443 | 0.05136 | 0.45 |
| Residuals | 38 | 0.70488 | 0.03916 | 0.92426 |  |  | 1.70201 | 0.094556 | 0.94864 |  |  |
| Total | 39 | 0.76264 | 1 |  |  |  | 1.79415 | 1 |  |  |  |
|  | Df | SumsOfSqs | MeanSqs | F.Model | R2 | Pr(>F) | SumsOfSqs | MeanSqs | F.Model | R2 | Pr(>F) |
| Prevotella.copri.MGS042 | 1 | 0.04434 | 0.044343 | 1.1112 | 0.05814 | 0.347 | 0.08915 | 0.089153 | 0.9412 | 0.04969 | 0.474 |
| Residuals | 38 | 0.71829 | 0.039905 | 0.94186 |  |  | 1.705 | 0.094722 | 0.95031 |  |  |
| Total | 39 | 0.76264 | 1 |  |  |  | 1.79415 | 1 |  |  |  |
|  | Df | SumsOfSqs | MeanSqs | F.Model | R2 | Pr(>F) | SumsOfSqs | MeanSqs | F.Model | R2 | Pr(>F) |
| Prevotella.copri.MGS046 | 1 | 0.05345 | 0.053445 | 1.3565 | 0.07008 | 0.224 | 0.11271 | 0.112709 | 1.2066 | 0.06282 | 0.268 |
| Residuals | 38 | 0.70919 | 0.039399 | 0.92992 |  |  | 1.68144 | 0.093413 | 0.93718 |  |  |
| Total | 39 | 0.76264 | 1 |  |  |  | 1.79415 | 1 |  |  |  |
|  | Df | SumsOfSqs | MeanSqs | F.Model | R2 | Pr(>F) | SumsOfSqs | MeanSqs | F.Model | R2 | Pr(>F) |
| Prevotella.copri.MGS050 | 1 | 0.05993 | 0.059927 | 1.535 | 0.07858 | 0.169 | 0.23393 | 0.233931 | 2.6988 | 0.13039 | 0.005 |
| Residuals | 38 | 0.70271 | 0.039039 | 0.92142 |  |  | 1.56022 | 0.086679 | 0.86961 |  |  |
| Total | 39 | 0.76264 | 1 |  |  |  | 1.79415 | 1 |  |  |  |
|  | Df | SumsOfSqs | MeanSqs | F.Model | R2 | Pr(>F) | SumsOfSqs | MeanSqs | F.Model | R2 | Pr(>F) |
| Prevotella.copri.MGS051 | 1 | 0.06123 | 0.061229 | 1.5713 | 0.08029 | 0.155 | 0.19737 | 0.19737 | 2.2248 | 0.11 | 0.02 |
| Residuals | 38 | 0.70141 | 0.038967 | 0.91971 |  |  | 1.59679 | 0.08871 | 0.89 |  |  |
| Total | 39 | 0.76264 | 1 |  |  |  | 1.79415 | 1 |  |  |  |
|  | Df | SumsOfSqs | MeanSqs | F.Model | R2 | Pr(>F) | SumsOfSqs | MeanSqs | F.Model | R2 | Pr(>F) |
| Roseburia.intestinalis.MGS026 | 1 | 0.05246 | 0.052459 | 1.3296 | 0.06879 | 0.212 | 0.114 | 0.114005 | 1.2214 | 0.06354 | 0.266 |
| Residuals | 38 | 0.71018 | 0.039454 | 0.93121 |  |  | 1.6802 | 0.093341 | 0.93646 |  |  |
| Total | 39 | 0.76264 | 1 |  |  |  | 1.7941 | 1 |  |  |  |
|  | Df | SumsOfSqs | MeanSqs | F.Model | R2 | Pr(>F) | SumsOfSqs | MeanSqs | F.Model | R2 | Pr(>F) |
| Roseburia.inulinivorans.MGS029 | 1 | 0.01554 | 0.015539 | 0.37439 | 0.02038 | 0.896 | 0.06802 | 0.068024 | 0.70935 | 0.03791 | 0.71 |
| Residuals | 38 | 0.7471 | 0.041505 | 0.97962 |  |  | 1.72613 | 0.095896 | 0.96209 |  |  |
| Total | 39 | 0.76264 | 1 |  |  |  | 1.79415 | 1 |  |  |  |
|  | Df | SumsOfSqs | MeanSqs | F.Model | R2 | Pr(>F) | SumsOfSqs | MeanSqs | F.Model | R2 | Pr(>F) |
| Roseburia.inulinivorans.MGS148 | 1 | 0.21291 | 0.212906 | 6.9713 | 0.27917 | 0.003 | 0.21882 | 0.218822 | 2.5003 | 0.12196 | 0.006 |
| Residuals | 38 | 0.54973 | 0.030541 | 0.72083 |  |  | 1.57533 | 0.087518 | 0.87804 |  |  |
| Total | 39 | 0.76264 | 1 |  |  |  | 1.79415 | 1 |  |  |  |
|  | Df | SumsOfSqs | MeanSqs | F.Model | R2 | Pr(>F) | SumsOfSqs | MeanSqs | F.Model | R2 | Pr(>F) |
| Subdoligranulum.variabile.MGS011 | 1 | 0.15027 | 0.15027 | 4.4172 | 0.19705 | 0.003 | 0.12725 | 0.127246 | 1.3741 | 0.07092 | 0.189 |
| Residuals | 38 | 0.61236 | 0.03402 | 0.80295 |  |  | 1.66691 | 0.092606 | 0.92908 |  |  |
| Total | 39 | 0.76264 | 1 |  |  |  | 1.79415 | 1 |  |  |  |
|  | Df | SumsOfSqs | MeanSqs | F.Model | R2 | Pr(>F) | SumsOfSqs | MeanSqs | F.Model | R2 | Pr(>F) |
| Subdoligranulum.variabile.MGS028 | 1 | 0.06057 | 0.060567 | 1.5529 | 0.07942 | 0.157 | 0.17383 | 0.173826 | 1.931 | 0.09688 | 0.046 |
| Residuals | 38 | 0.70207 | 0.039004 | 0.92058 |  |  | 1.62033 | 0.090018 | 0.90312 |  |  |
| Total | 39 | 0.76264 | 1 |  |  |  | 1.79415 | 1 |  |  |  |
|  | Df | SumsOfSqs | MeanSqs | F.Model | R2 | Pr(>F) | SumsOfSqs | MeanSqs | F.Model | R2 | Pr(>F) |
| Subdoligranulum.variabile.MGS031 | 1 | 0.07054 | 0.070535 | 1.8345 | 0.09249 | 0.1 | 0.24806 | 0.248063 | 2.888 | 0.13826 | 0.002 |
| Residuals | 38 | 0.6921 | 0.03845 | 0.90751 |  |  | 1.54609 | 0.085894 | 0.86174 |  |  |
| Total | 39 | 0.76264 | 1 |  |  |  | 1.79415 | 1 |  |  |  |
